# Tumor acidification and GSH depletion by bimetallic composite nanoparticles for enhanced chemodynamic therapy of TNBC

**DOI:** 10.1186/s12951-024-02308-8

**Published:** 2024-03-09

**Authors:** Wenting Chen, Fangfang Hu, Qian Gao, Caiyun Zheng, Que Bai, Jinxi Liu, Na Sun, Wenhui Zhang, Yanni Zhang, Kai Dong, Tingli Lu

**Affiliations:** 1 Key Laboratory of Space Bioscience and Biotechnology, Engineering Research Center of Chinese Ministry of Education for Biological Diagnosis, Treatment and Protection Technology and Equipment, School of Life Sciences, Northwestern Polytechnical University, No. 127 West Youyi Road, Xi’an, 710072 People’s Republic of China; 2 Frontiers Science Center for Flexible Electronics, Xi’an Institute of Flexible Electronics and Xi’an Institute of Biomedical Materials & Engineering, Northwestern Polytechnical University, No. 127 West Youyi Road, Xi’an, 710072 People’s Republic of China; 3 School of Pharmacy, Xi’an Jiaotong University, No. 76 Yanta West Road, Xi’an 710061, People’s Republic of China

**Keywords:** Chemodynamic therapy, Reactive oxygen species, Gallic acid, Fenton reaction, Metal-polyphenol networks

## Abstract

Chemodynamic therapy (CDT) based on intracellular Fenton reaction to produce highly cytotoxic reactive oxygen species (ROS) has played an essential role in tumor therapy. However, this therapy still needs to be improved by weakly acidic pH and over-expression of glutathione (GSH) in tumor microenvironment (TEM), which hinders its future application. Herein, we reported a multifunctional bimetallic composite nanoparticle MnO_2_@GA-Fe@CAI based on a metal polyphenol network (MPN) structure, which could reduce intracellular pH and endogenous GSH by remodeling tumor microenvironment to improve Fenton activity. MnO_2_ nanoparticles were prepared first and MnO_2_@GA-Fe nanoparticles with Fe^3+^ as central ion and gallic acid (GA) as surface ligands were prepared by the chelation reaction. Then, carbonic anhydrase inhibitor (CAI) was coupled with GA to form MnO_2_@GA-Fe@CAI. The properties of the bimetallic composite nanoparticles were studied, and the results showed that CAI could reduce intracellular pH. At the same time, MnO_2_ could deplete intracellular GSH and produce Mn^2+^ via redox reactions, which re-established the TME with low pH and GSH. In addition, GA reduced Fe^3+^ to Fe^2+^. Mn^2+^ and Fe^2+^ catalyzed the endogenous H_2_O_2_ to produce high-lever ROS to kill tumor cells. Compared with MnO_2_, MnO_2_@GA-Fe@CAI could reduce the tumor weight and volume for the xenograft MDA-MB-231 tumor-bearing mice and the final tumor inhibition rate of 58.09 ± 5.77%, showing the improved therapeutic effect as well as the biological safety. Therefore, this study achieved the high-efficiency CDT effect catalyzed by bimetallic through reshaping the tumor microenvironment.

**Graphical Abstract:**

**Figure:**
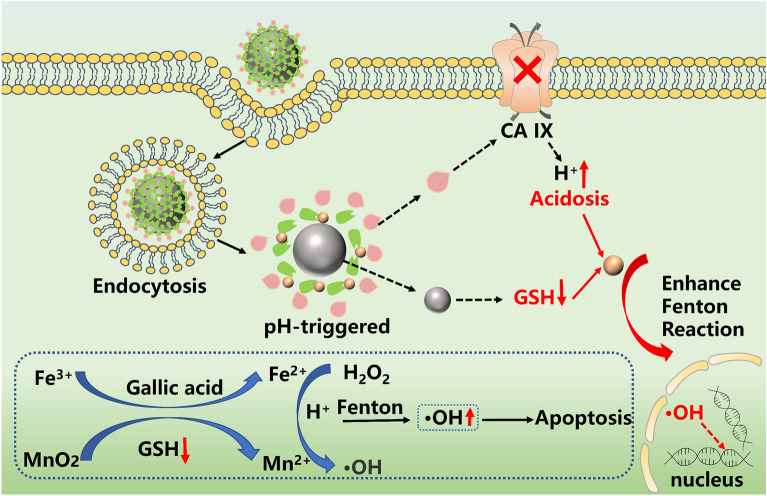

**Supplementary Information:**

The online version contains supplementary material available at 10.1186/s12951-024-02308-8.

## Introduction

Triple-negative breast cancer (TNBC) is a specific subtype of cancer characterized by high expression of carbonic anhydrase IX (CA IX) on the cell membrane, particularly under hypoxic conditions. This increased expression of CA IX contributes to the aggressive and poor prognostic features associated with TNBC [1–3]. However, due to the absence of specific therapeutic targets, treatment for TNBC remains suboptimal. Chemodynamic therapy (CDT) has recently emerged as a promising tumor treatment approach [4–6]. The basic principle of CDT is based on the relatively low pH and high endogenous H_2_O_2_ in the tumor microenvironment (TME), using transition metal ions as catalysts to react with H_2_O_2_ in the acidic TME to produce hydroxyl radicals (·OH) to kill tumor cells [7–9]. However, the efficiency of CDT is limited by the physical tumor microenvironment, such as the high expression level of GSH and the weakly acidic pH, leading to reduced catalytic efficiency [10–14]. Therefore, it is crucial to investigate effective strategies for intracellular acidification and GSH depletion to enhance CDT efficacy.

The over-expression of intracellular glutathione (GSH) in tumor cells is considered a primary barrier to ROS-related treatments [15–17]. Effective reduction of GSH levels has been identified as a viable strategy for disrupting intracellular redox homeostasis. Low GSH levels render cancer cells more vulnerable to external oxidative stress and chemotherapy drugs [18, 19]. Related studies have shown that GSH scavengers can effectively reduce GSH overexpression at tumor sites and enhance the efficacy of CDT [20, 21]. Furthermore, the acidic TME plays a crucial role in CDT [22, 23]. Carbon dioxide (CO_2_) serves as the primary source of acidity within the tumor, and CA IX catalyzes the conversion of CO_2_ to bicarbonate (HCO_3_^−^) and protons (H^+^) (CO_2_ + H_2_O ⇋ HCO_3_^−^  + H^+^) [24, 25]. Under hypoxic conditions, the accumulation of hypoxia-inducible factor-1α (HIF-1α) may further induce high expression of CA IX, leading to various downstream effects, including extracellular acidification, loss of cell adhesion, and increased tumor cell migration [26]. Therefore, using proton pump inhibitors targeting tumor pH regulation proteins holds promise for modulating proton pump activity, inhibiting lactate proton efflux, and inducing intracellular acidification.

Numerous recent experiments have revealed the apoptosis-promoting effects of metal polyphenol networks (MPNs) in tumor treatment [27–29]. The main advantage of MPN as a therapeutic strategy for tumors is the redox difference between cancer cells and normal cells, which helps polyphenols to further target tumor cells [30, 31]. Polyphenols exhibit exceptional biocompatibility, sensitivity to acidity, and modifiability, thereby promoting the Fenton/Fenton-like reaction and accelerating the transformation between Fe^3+^ and Fe^2+^ [32]. Currently, several polyphenols have received FDA approval for applications in food preparation and human health protection [33]. Gallic acid (GA), a naturally occurring phenolic antioxidant found in plants and various food sources, such as green tea and grapes, exhibits remarkable anti-tumor, antioxidant, and anti-inflammatory properties [34–36]. GA can combine with Fe^3+^ through coordination chelation and continuously reduce Fe^3+^ to Fe^2+^, further enhancing the catalytic stability of Fe^2+^ under physiological conditions.

**Scheme 1:**
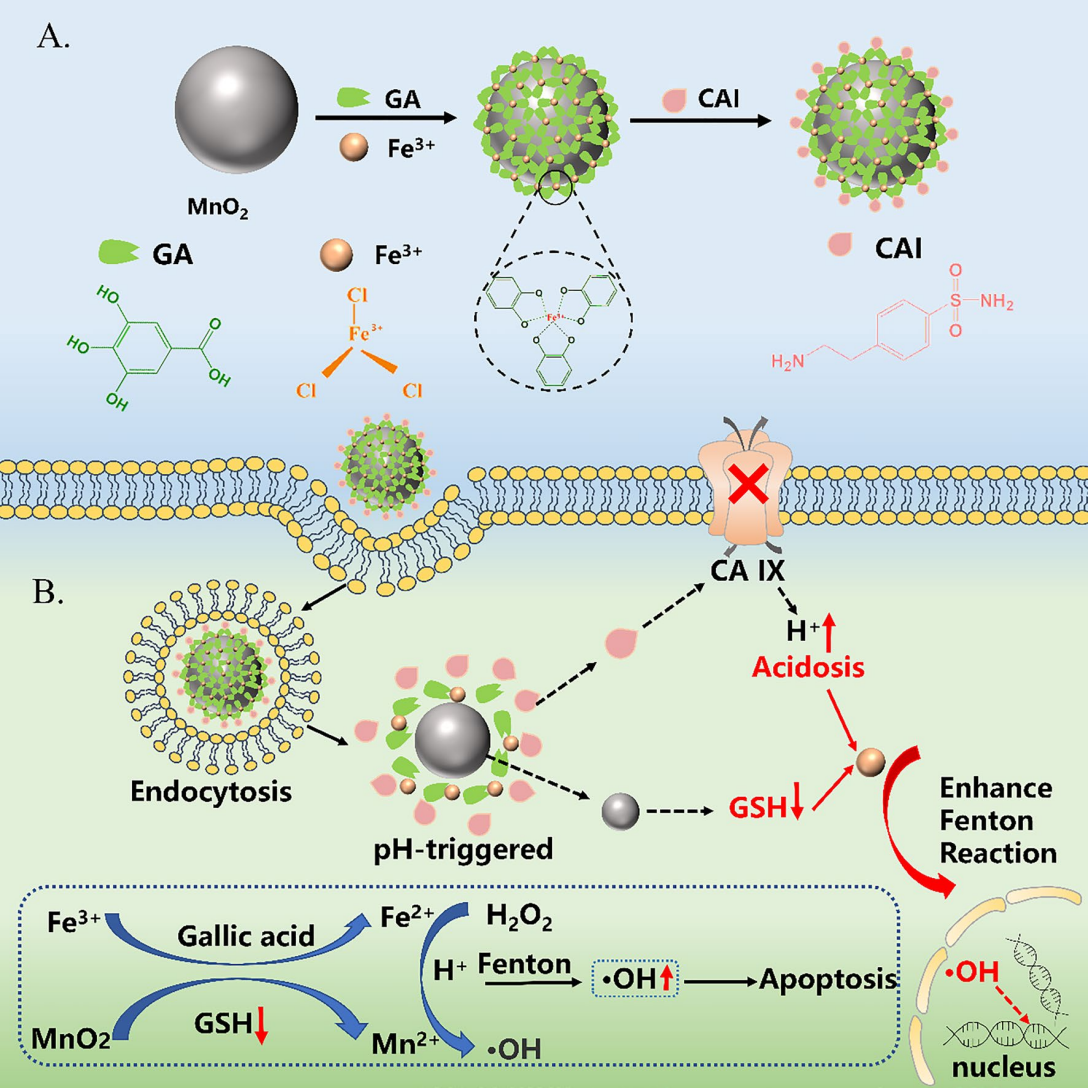
**A**. Preparation of MnO_2_@GA-Fe@CAI nanoparticles. **B**. Schematic diagram of CDT enhancement by bimetallic MnO_2_@GA-Fe@CAI nanoparticles modulating the tumor microenvironment

Herein, we design a multifunctional bimetallic composite nanoparticle MnO_2_@GA-Fe@CAI with the structure of a metal polyphenol network. First, MnO_2_ nanoparticles are prepared and MnO_2_@GA-Fe is formed by chelation reaction with Fe^3+^ as its central ion and GA as the outer ligand. Then, MnO_2_@GA-Fe@CAI are obtained by coupling the carboxyl group of GA with 4-(2-aminoethyl) benzenesulfonamide (abbreviated as the CA IX inhibitor, CAI) by amide reaction (Scheme 1A). In theory, MnO_2_@GA-Fe@CAI nanoparticles can enter tumor cells, inhibit the activity and expression of CA IX and reduce the pH value of TME, which is favorable for the Fenton reaction. After the nanoparticles are taken by the tumor cell, due to the weak acidic pH in TME, the MPN structure gradually depolymerizes, and MnO_2_ converts GSH to GSSG, which can reduce the content of antioxidant substances in the tumor cells (MnO_2_ + 2GSH + 2H^+^  → Mn^2+^  + GSSG + 2H_2_O). Meanwhile, Mn^4+^ is reduced to Mn^2+^ and the free GA reduces Fe^3+^ to Fe^2+^. Ultimately, Mn^2+^ and Fe^2+^ can act as catalysts in the Fenton reaction to synergistically catalyze the production of ·OH from H_2_O_2_, realizing efficient synergistic treatment of TNBC (Scheme 1B).

## Materials and methods

### Materials

Potassium permanganate (KMnO_4_), polyvinyl pyrrolidone (PVP), methylene blue (MB), 3,3′,5,5′ -tetramethylbenzidine (TMB), hydrogen peroxide (H_2_O_2_), and triethylamine (TEA) were purchased from the Fuchen Chemical Reagent Co., Ltd. (Tianjin, China). Poly (allylamine hydrochloride) (PAH, 15 kDa) was obtained from Wokai Biotechnology Co., Ltd. (Beijing, China). Gallic acid (GA), 4-(2-aminoethyl) benzenesulfonamide (abbreviated as the CA IX inhibitor, CAI), and sodium hydroxide (NaOH) were purchased from the Sigma-Aldrich, Inc. (St Louis, MO, USA). Fe^3+^ chloride anhydrous (FeCl_3_), 1-(3-Dimethylaminopropyl)-3-ethyl carbodiimide hydrochloride (EDC·HCl) were purchased from the Aladdin Chemistry Co., Ltd. (Shanghai, China). Fetal bovine serum (FBS) and high glucose Dulbecco’s modified Eagle’s medium (DMEM) were purchased from Hyclone (USA). Cell counting kit-8 (CCK-8), ROS assay kit (DCFH-DA), Annexin V-EGFP Apoptosis Detection Kit, calcein-AM/Propidium Iodide (PI) assay kit, Hoechst 33342, GSH and GSSG Assay Kit, enhanced mitochondrial membrane potential assay kit (JC-1) and the intracellular pH fluorescence probe (BCECF-AM) were obtained from Beyotime Biotechnology (Shanghai, China). All other reagents were analytical grade.

MDA-MB-231 cells, the human breast carcinoma cell line (STR-identified correct), were purchased from Wuhan Procell Life Science&Technology Co., Ltd. L929 cells, mouse fibroblasts (STR-identified correct), were purchased from the Cell Bank of Typical Culture Collection Committee of Chinese Academy of Sciences. The cell was cultured separated under normoxia (37 ℃, 5% CO_2_) and hypoxia (37 ℃, 1% O_2_, 5% CO_2,_ and 95% N_2_). Cells were cultured in DMEM with 10% FBS, penicillin (100 units/mL), and streptomycin (100 mg/mL).

### Characterization

Transmission electron microscope (TEM, Talos F200X, FEI, USA) was used to examine the internal morphology of the samples. The elemental composition of nanoparticles was analyzed by X-ray energy dispersive spectroscopy (EDS, Talos F200X, FEI, USA). The Fourier transform infrared spectroscopy (FTIR, FTIR-8400S, Shimadzu, Japan). X-ray photoelectron spectroscopy (XPS, ESCALAB Xi + , 191 Thermo Fisher, USA) was used to measure the samples’ chemical compositions and valence state. UV–vis absorption spectra were collected with a spectrophotometer (UV- 2600, Japan). The samples' particle size and zeta potential were measured by laser particle size and zeta potential meter (Zeta; Malvern, UK). The content of Mn and Fe in MnO_2_@GA-Fe@CAI was measured by an inductively coupled plasma optical emission spectrometer (ICP-OES, Agilent 5110, USA).

### The synthesis of MnO_2_

MnO_2_ nanoparticles were prepared based on the redox reaction of PAH with KMnO_4_ [37]. 2.0 mL aqueous solution of PAH (37.4 mg/mL) was added dropwise into 18 mL aqueous solution of KMnO_4_ (3.5 mg/mL) and stirred at room temperature for 15 min until all the purple solution turned dark brown. The MnO_2_ solution was then dialyzed (MW: 3000 Da) for 24 h and freeze-dried.

### The synthesis of MnO_2_@GA-Fe@CAI

First, MnO_2_@GA-Fe was synthesized as follows. 1.0 mg MnO_2_ was dispersed into 1 mL of ultrapure water, followed by PVP (1 mL, 10 mg/mL) and 1 mL of GA solution (3 mg/mL in ultrapure water). Then, 20 μL of TEA was added and stirred (400 rpm for 5 min). Afterward, FeCl_3_ (1 mL, 6 mg/mL) was added and stirred at room temperature (400 rpm for 24 h). The final product was dialyzed overnight (MW: 8000 ~ 1300 Da) and stored at 4 ℃  for further experiments.

MnO_2_@GA-Fe@CAI was further synthesized through an amide reaction. Briefly, 5 mL of CAI (15 mg/mL) was added dropwise into 5 mL of MnO_2_@GA-Fe aqueous solution (15 mg/mL) and sonicated for 30 min. EDC (5 mL, 5 mg/mL) was added and stirred at room temperature (400 rpm for 24 h, pH 8.0). The final product was placed in a dialysis bag (MW: 8000 ~ 1300 Da), dialyzed overnight, and freeze-dried.

### GSH responsiveness and consumption in vitro

The solution containing 200 μL GSH (0, 0.5, 1, 2, 4, 5, 10 mM) was respectively added dropwise to 150 μg/mL MnO_2_ solution, 9000 rpm for 3 min. The supernatant was detected by UV–vis absorption at 300 nm. In order to further investigate the ability of GSH consumption in vitro, different concentrations of MnO_2_@GA-Fe@CAI was mixed with GSH. After 30 min, the residual GSH was detected using5,5'-Dithio bis-(2-nitrobenzoic acid) (DTNB) as a probe. The yellow product from the reaction of DTNB with the thiol group (− SH) of GSH was recorded via UV–vis spectrophotometer at 412 nm.

### In vitro ROS generation detection

Degradation of methylene blue (MB). Equivalent MnO_2_@GA-Fe@CAI was incubated in NaHCO_3_ (25 mM) buffer containing different concentrations of GSH (0, 0.5, 1.0, 10 mM) for 15 min at 37 ℃. After centrifugation at 9000 rpm for 3 min, the supernatant was removed and 10 μg/mL of MB and 60 mM of H_2_O_2_ were added and shaken at 37 ℃ for 4 h. The change in the UV–vis absorption of MB at 665 nm was measured.

Oxidation of 3,3′,5,5′-tetramethylbenzidine (oxTMB). MnO_2_@GA-Fe@CAI solutions at different concentrations (250, 300, 350, 400, 450, 500 μg/mL) were mixed thoroughly with NaHCO_3_ (25 mM) solution containing 1.0 mM GSH for 15 min at 37 ℃. After centrifugation at 9000 rpm for 3 min, TMB (400 μM) and H_2_O_2_ (60 mM) were added to the supernatant. The absorbance of oxTMB at 450 nm at different times was measured using a microplate reader (ELX808, BioTek, USA).

### In vitro stability

1 mg/mL MnO_2_@GA-Fe@CAI nanoparticles were placed in different physiological environment solutions (ultrapure water, PBS, DMEM (with FBS)) and incubated in a shaker (37 ℃, 100 rpm) to observe their stability. Subsequently, the supernatants were taken from the solutions at different time points (0, 2, 4, 6, 8, 12, and 24 h), and the size and PDI of nanoparticles were measured by DLS.

### In vitro Mn^2+^ and Fe^2+^ release

5 mL MnO_2_@GA-Fe CAI (3 mg/mL) was dialyzed at pH of 5.0, 6.5, and 7.4 in 30 mL ultrapure water, respectively. At predetermined time points (10, 30 min, 1, 2, 4, 8, 12, 24 h), 3 mL of fluid outside the dialysis bag was removed and replenished with an equal temperature and volume of release medium. Finally, the Mn^2+^ and Fe^2+^ content was measured by ICP-OES.

### Cellular experiments

#### Cytocompatibility of nanoparticles

The cytotoxicity of nanoparticles on L929 and MDA-MB-231 cells was assessed by CCK-8 assay. Take L929 for example. L929 was seeded with a density of 5 × 10^3^ per well in 96-well plates for 24 h under normoxia and hypoxia, respectively. Then, cells were co-incubated with DMEM complete medium containing MnO_2_, MnO_2_@GA-Fe, and MnO_2_@GA-Fe@CAI nanoparticles (100 μL) and placed under normoxia for 24 h. Nanoparticle concentrations were 6.25, 12.5, 25, 50, 75, and 100 μg/mL (based on Fe concentration) and MnO_2_ was converted according to wt% (Mn: Fe = 1:10). The cytocompatibility of nanoparticles to cells under hypoxia was also tested following the above method. Normoxia: 37 ℃, 5% CO_2_; Hypoxia: 37 ℃, 1% O_2_, 5% CO_2_, and 95% N_2_. The cell culture medium used in both groups was DMEM, a high-glucose complete culture medium.

#### Living and death staining

The cytotoxicity of nanoparticles was further studied by the calcein-AM/PI stain of live/dead cells. Firstly, cells were co-incubated with different nanoparticles (the concentration was 100 μg/mL according to the Fe concentration) in a 96-well plate for 24 h under normoxia and hypoxia, respectively. Then, each group of cells was stained with calcein AM (green: live cells) and PI (red: dead cells) for 30 min. Finally, the cells were observed under an inverted fluorescence microscope (DMIL, Leica, Germany).

#### In vitro hemolysis assay

The hemolytic activity of the nanoparticles was assessed using the post-dilution whole-blood method. Firstly, 2 mL of rat blood was added to 2.5 mL PBS. 200 μL diluted blood was added into 800 μL PBS with different concentrations of nanoparticles (0.0625, 0.125, 0.25, 0.5, 1 mg/mL), mixed thoroughly on a shaker at 37 ℃ for 1 h, and photographed. Then, centrifuged at 3000 rpm for 10 min, the supernatant (100 μL) was transferred to a 96-well plate. The absorbance at 540 nm was read using a microplate reader. The UV–vis absorption of the samples in 480 ~ 680 nm was also measured. Triton X-100 and PBS were used as positive and negative controls. Finally, the hemolysis rate was calculated by the following formula:

$$\text{Hemolysis ratio(\%)=}\frac{{OD}_{e}-{OD}_{p}}{{OD}_{t}-{OD}_{p}}\times \text{100\%}$$

ODe: the absorbance value of the experimental group, ODt: the absorbance of the positive control (Triton X-100), ODp: absorbance value of the negative control (PBS).

#### Detection of intracellular ROS

Intracellular ROS production was detected using the Reactive Oxygen Species Assay Kit (ROS Assay Kit) and Hoechst 33342. MDA-MB-231 inoculated in a 6-well plate were co-incubated with media containing MnO_2_, MnO_2_@GA-Fe, MnO_2_@GA-Fe@CAI nanoparticles (at a concentration of 100 μg/mL depending on the Fe concentration) under normoxia and hypoxia for 12 h, respectively. Each group was treated with a DCFH-DA probe for 20 min, washed 3 times with PBS, and fixed with paraformaldehyde (4%, 1 mL) for 15 min. Subsequently, the cell nucleus was re-stained with Hoechst 333421 (1 mL) for 5 min and washed 3 times with PBS. ROS production was observed by inverted fluorescence microscopy (DMIL, Leica, Germany). Image J software (National Institutes of Health, USA) was used for semi-quantitative fluorescence analysis in each image.

#### Cell apoptosis test

The Annexin V-EGFP Apoptosis Detection Kit was used to detect the effect of nanoparticles on the apoptosis of MDA-MB-231. There were 7 groups: blank, PI, Annexin V-EGFP, MnO_2_ + Annexin V-EGFP + PI, MnO_2_@GA-Fe + Annexin V-EGFP + PI, and MnO_2_@GA-Fe@CAI + Annexin V-EGFP + PI. Finally, flow cytometry was performed and Annexin V-EGFP was green fluorescence detected by FL1 and PI was red fluorescence detected by FL3 channel (Excitation wavelength: Ex = 488 nm; Emission wavelength: Em: 530 nm).

#### Mitochondrial membrane potential detection by JC—1

MDA-MB-231 were seeded with a density of 1.0 × 10^5^ per well in a 24-well plate and cultured for 24 h under normoxia and hypoxia, respectively. Cells were co-incubated with nanoparticles at 100 μg/mL concentration according to the Fe content for 12 h. Then, the cells were stained with JC-1 and analyzed by an inverted fluorescence microscope. Image fluorescence was analyzed using Image J software. The results were expressed as the average JC-1 red/green (FL2/FL1) signal intensity ratio.

#### GSH/GSSG test

The GSH/GSSG ratio was analyzed using GSH and GSSG assay kits. The experimental protocol was as follows: 1.0 mL MDA-MB-231 were inoculated in 24-well plates at a density of 1.0 × 10^5^ cells/well and incubated in normoxia and hypoxia at 37 ℃ for 24 h. The medium was discarded and 1.0 mL of complete medium was added to the control group; the experimental group was added with complete medium containing (MnO_2_, MnO_2_@GA-Fe, MnO_2_@GA-Fe@CAI). The amount of MnO_2_ would be converted based on the ICP-OES results of Mn elemental content wt% (Mn:Fe = 1:10). The samples were incubated for 12 h in normoxia and hypoxia. There were 6 parallel groups were set up. (3 groups were used to determine the total glutathione content and 3 groups were used to measure the oxidized GSH content). The final results were determined by enzyme marker.

#### Cell migration and invasion test

MDA-MB-231 was treated with serum-free medium containing MnO_2_, MnO_2_@GA-Fe, and MnO_2_@GA-Fe@CAI (based on Fe concentration of 100 μg/mL) and incubated under hypoxia and normoxia for 12 h. A transwell of 8 μm was added with Matrigel (30 μL) diluted in a serum-free medium. Matrigel was incubated at 37 ℃ for 120 min to polymerize into a gel (Matrigel was not added to the upper chamber of the transwell for migration experiments). The cell suspension (1.0 × 10^4^ cells/well, 100 μL) was then inoculated into the transwell chambers, and 500 μL of medium (containing 20% FBS) was added to the lower chamber. Finally, the medium was carefully removed, the remaining cells in the upper chamber were removed by wiping with a cotton swab, and the cells on the lower surface of the transwell were fixed in 4% paraformaldehyde for 15 min, followed by staining with 0.2% crystal violet for 10 min and washing three times with PBS to observe migration and invasion by inverted fluorescence microscope (DMIL, Leica, Germany).

#### Intracellular H^+^ accumulation assay

MDA-MB-231 were seeded with a density of 1.0 × 10^5^ per well in a 24-well plate and cultured for 24 h under normoxia and hypoxia, respectively. Cells were co-incubated with nanoparticles at 100 μg/mL concentration according to the Fe concentration for 12 h. Then, the cells were stained with BCECF-AM and analyzed by an inverted fluorescence microscope. Image fluorescence analysis was done using Image J software (National Institutes of Health, USA).

#### In vivo anti-cancer performance

In vivo experiments were carried out using Balb/c-nude mice (4 weeks, 18 ~ 19 g, 5 mice per group) purchased from Keao Biotechnology Co., Ltd. (Xi'an, China). All procedures were approved by the Institutional Animal Care and Use Committee (IACUC) of Northwestern Polytechnical University.

Firstly, MDA-MB-231 cell (1 × 10^7^ cells/site, 200 μL dose for each mouse) was implanted subcutaneously in the right axilla of mice. Mice were continuously observed and the volume of tumors was monitored. When the volume of tumors reached 0.1 cm^3^, MDA-MB-231 tumor-bearing mice were randomly divided into 4 groups, with 5 mice in each. The mice in the control group were injected intratumorally (i.t) with the saline solution. The mice in the other three groups were treated with MnO_2,_ MnO_2_@GA-Fe, and MnO_2_@GA-Fe@CAI nanoparticles, respectively. Different formulations were given intratumorally on days 0, 4, 8 and 12 d. The injection dose for the above group was set at 5 mg/kg (50 μL) according to the concentration of Fe in each nanoparticle. Tumor volume was monitored using vernier calipers every 2 days lasted for 21 days and was calculated by the following equation:

$$\text{TV} = \frac{1}{2}\times a\times {b}^{2}$$

Here, a: the tumor major axis, and b: the tumor minor axis.

After 21 d, the tumor tissue and the main organs of the mice (heart, liver, spleen, lung and kidney) were collected and subsequently fixed in 4% paraformaldehyde for 24 h. H&E, TUNEL and TNF-α staining were performed on tumor tissues to evaluate the CDT anti-tumor activity of different nanoparticles. H&E staining was also performed on major organs.

In order to understand the distribution of MnO_2_@GA-Fe@CAI in the mice, we employed MnO_2_@GA-Fe@CAI loaded with a fluorescent probe, 1,1-dioctadecyl-l-3,3,3,3-tetramethylindodicarbocyanine perchlorate (DiD), as a model nanoparticle. Firstly, mice were injected intratumorally (i.t) with DiD-MnO_2_@GA-Fe@CAI. Then, the fluorescence imaging of mice was performed by IVIS at different time points (0, 3, 6, 12 and 24 h) after administration. After 24 h, the distribution of nanoparticles in major organs and tumors was observed in vitro. The excitation wavelength was set at 640 nm, and the emission wavelength was set at 670 nm.

#### Statistical analysis

One-way analysis of variance (ANOVA) was used for statistical analysis to determine the statistical difference between groups. Graph Pad Prism 8.0 was used for statistical analysis. Results were represented as mean ± standard deviation (SD). P < 0.05 was statistically significant. There were at least 3 parallel samples for each group. (n = 3, * p < 0.05, ** *p* < 0.01 and *** p < 0.001).

## Results and discussion

### Synthesis and characterization of MnO_2_@GA-Fe@CAI

**Fig. 1:**
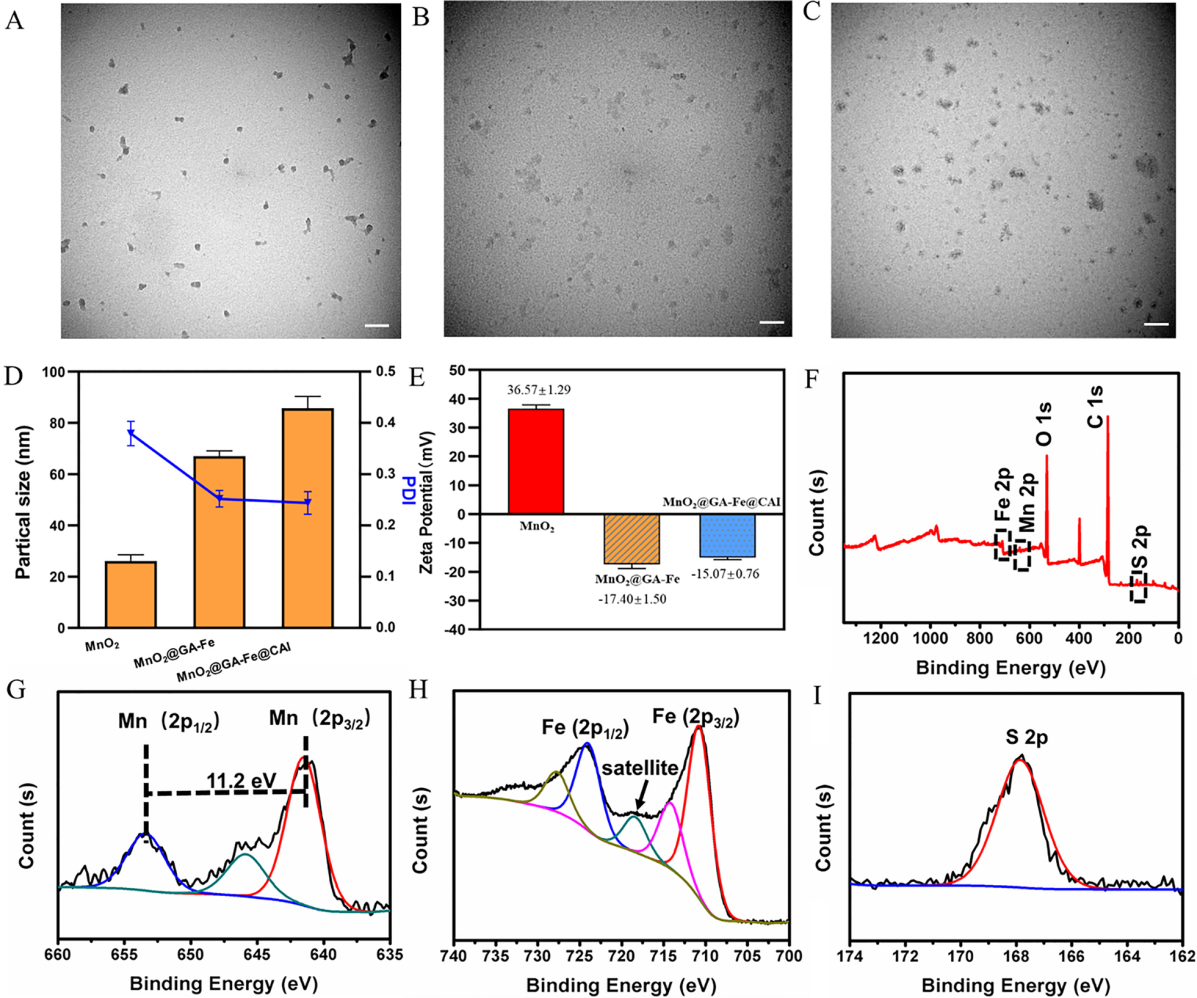
TEM image of nanoparticles (**A**) MnO_2_ (**B**) MnO_2_@GA-Fe (**C**) MnO_2_@GA-Fe@CAI (scale: 100 nm). **D** Particle size and PDI of nanoparticles. **E** Zeta potential of nanoparticles. **F** XPS full spectrum, (**G**) Mn 2p, (**H**) Fe 2p, and (**I**) S 2p of MnO_2_@GA-Fe@CAI

MnO_2_@GA-Fe@CAI nanoparticle was synthesized and TEM images showed it was spherical in a good decentralized state with an average size of 81.48 nm (Fig. 1A–C). Elemental analysis by energy dispersive X-ray spectroscopy (EDX) showed that the Fe, Mn, S, and O element were uniformly distributed in MnO_2_@GA-Fe@CAI (Additional file 1: Fig. S1A, B). Figure 1D shows the size and polydispersity index of three nanoparticles, and the further modification of GA-Fe and CAI made the size of MnO_2_ gradually increase with good dispersibility. The potential of MnO_2_@GA-Fe@CAI was − 15.07 ± 0.76 mV, and the negative charge was mainly attributed to GA introduced on the surface (Fig. 1E).

The full spectrum of MnO_2_@GA-Fe@CAI XPS is shown in Fig. 1F, where the typical peaks of C, O, N, Mn, Fe, and S elements were observed. Mn 2p1/2 and Mn 2p3/2 orbital peaks were located at 653.8 and 642.6 eV, and had a spin energy separation with 11.2 eV, which confirmed the obtained MnO_2_ nanoparticles (Fig. 1G). Fe 2p XPS was displayed at the feature peak 708.9 and 724.5 eV, which indicated that there was assigned to Fe^3+^ (Fig. 1H). The S 2p peak was located at 167.7 eV, which is attributed to the S element of 4-(2-aminoethyl) benzenesulfonamide (Fig. 1I). In addition, the analysis results of ICP-MS showed that the content of Mn and Fe in MnO_2_@GA-Fe@CAI was about 2.06% and 27.66%, respectively.

To further study the chemical structure of nanoparticles, their FTIR spectra were shown in Additional file 1: Fig. S2. For MnO_2_@GA-Fe@CAI, the peak at 1250 cm^−1^ (HO-C stretch band) was lower than that of GA due to the covalent bond formed by chelation between phenolic hydroxyl and metal ions. The stretching vibration peaks at 1680 cm^−1^ was the amide bond formed between the reaction of 4-(2-aminoethyl) benzamide and GA. In addition, the change in the UV–vis absorption spectrum of nanoparticles was also observed (Additional file 1: Fig. S3A, B). The absorption peak of benzenesulfonamide appeared at 252.5 nm in MnO_2_@GA-Fe@CAI, which further indicated the successful synthesis of MnO_2_@GA-Fe@CAI.

### GSH response and ·OH generation of MnO_2_@GA-Fe@CAI

**Fig. 2:**
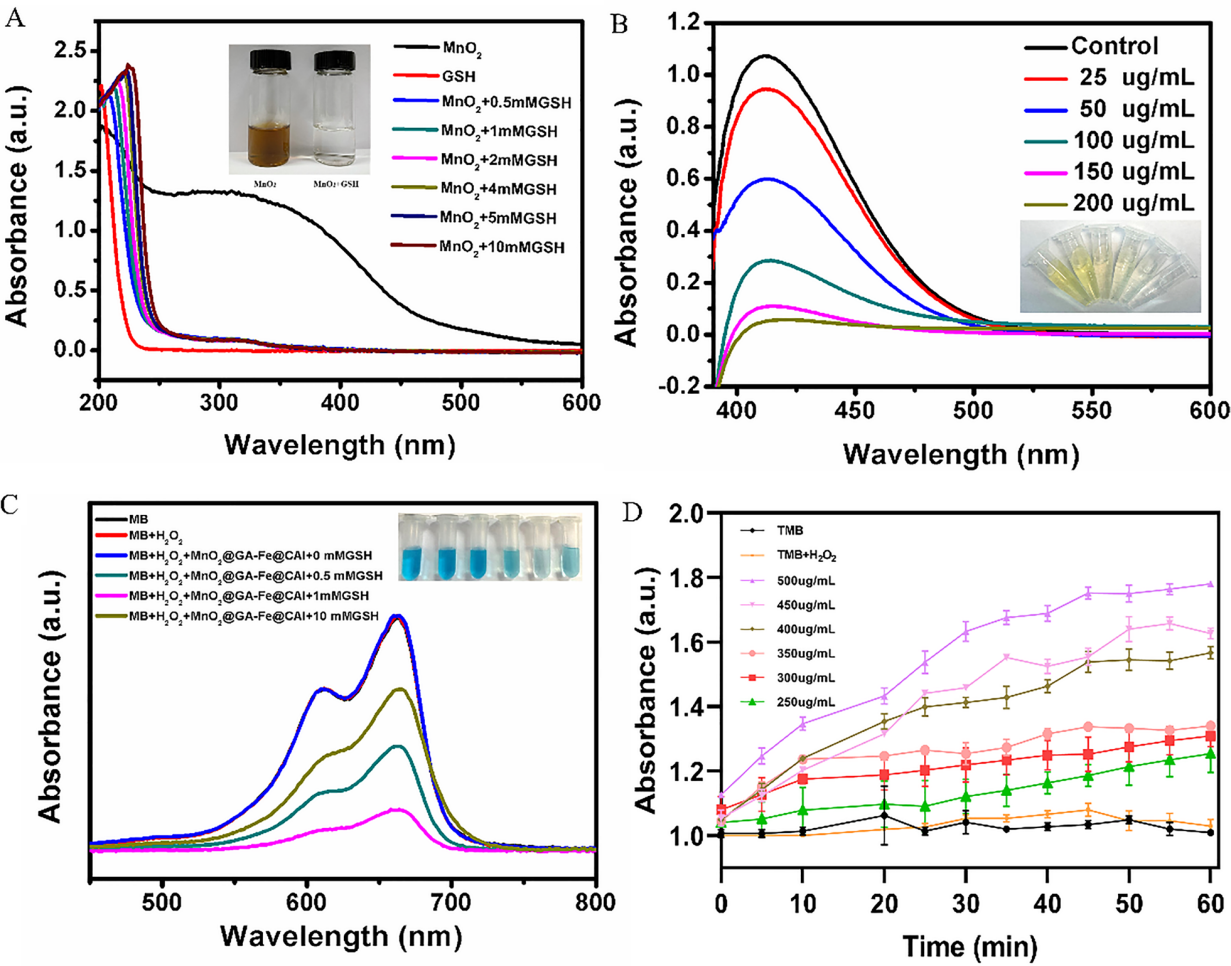
The properties of MnO_2_ and MnO_2_@GA-Fe@CAI in vitro. **A** UV–vis absorption spectra change of MnO_2_. **B** GSH consumption with different concentrations of MnO_2_@GA-Fe@CAI. **C** UV–vis absorption spectra of MnO_2_@GA-Fe@CAI and photographs (inset) of MB solution after different treatments. (Inset: from left to right, MB, MB + H_2_O_2_, MB + H_2_O_2_ + MnO_2_@GA-Fe@CAI + GSH (concentrations of 0, 0.5, 1 and 10 mM, respectively)). **D** The absorbance of ·OH generation is based on the catalytic oxidation of MnO_2_@GA-Fe@CAI with TMB at 450 nm

The responsive ability of MnO_2_ solution to different concentrations of GSH was evaluated. After adding GSH, the UV–vis absorption spectra of MnO_2_ solution changed, and the solution changed from brown to colorless (Fig. 2A). The characteristic absorption peak of MnO_2_ at 300 nm disappeared with the addition of different concentrations of GSH, indicating that there might exist redox reactions between MnO_2_ and GSH. As shown in Fig. 2B, a decrease in the absorbance at 412 nm was observed as the concentration of MnO_2_@GA-Fe@CAI increased, indicating that MnO_2_@GA-Fe@CAI had the ability to consume GSH.

The ability of the nanoparticles to produce ·OH in vitro was assessed by detecting changes in the color of methylene blue (MB) and its UV absorption peak. As shown in Fig. 2C, the UV–vis absorption peaks and color changes of mixed solution with different GSH concentrations from 0 to 10 mM. In the range of 0–1 mM, the MB solution changed from the original dark blue color to light blue significantly, and the absorption peak at 665 nm in the UV-absorption peaks decreased, indicating the degradation reaction of MB. However, when the GSH concentration was 10 mM, the UV–vis absorption of the solution increased, which should be caused by excessive consumption of ·OH by GSH. In addition, 3,3',5,5'-Tetramethylbenzidine (TMB) was used as an indicator to assess the performance of MnO_2_@GA-Fe@CAI in generating OH. As shown in Fig. 2D, the absorbance values of oxidized TMB (OX-TMB) at 450 nm increased with time and showed some concentration dependence. The above results confirmed that MnO_2_@GA-Fe@CAI could respond to GSH and improve the chemodynamic effect in vitro.

### Dispersibility and release behavior of MnO_2_@GA-Fe@CAI

**Fig. 3:**
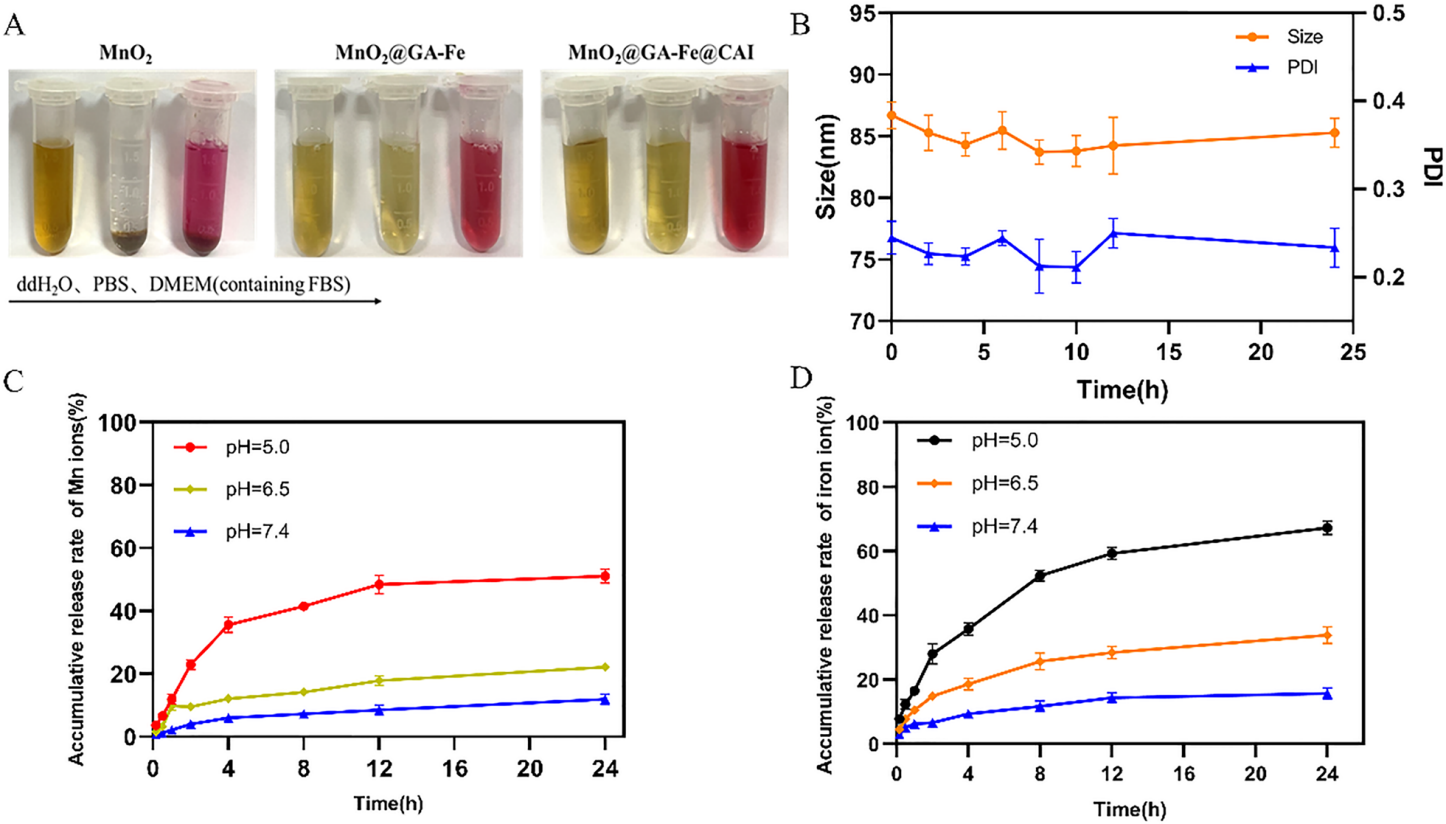
**A** Pictures of nanoparticles in different media (From left to right: ddH_2_O, PBS and DMEM containing 10% FBS). **B** Changes in particle size and PDI of MnO_2_@GA-Fe@CAI in PBS (pH = 7.4) for 24 h. Accumulative release curves of Mn^2+^ (**C**) and Fe^2+^ (**D**) in PBS with different pH values

The nanoparticles were dispersed in ddH_2_O, PBS (pH 7.4), and DMEM (containing FBS) for 24 h to evaluate their stability, and the results were shown in Fig. 3A. The polyelectrolyte-coated MnO_2_ were too small and positively charged; which caused them to exhibit poor dispersion in PBS or cellular media [37]. GA, as a negatively charged molecule, was linked on the MnO_2_ surface with phenolic hydroxyl groups, and it had good dispersion in the media without precipitation formation. The surface-modified nanoparticles showed almost no precipitation in the media and showed better dispersibility than MnO_2_. Additionally, the dispersion of MnO_2_@GA-Fe@CAI was tested in PBS (pH 7.4) for 24 h. The aliquot samples were withdrawn at predetermined time intervals and the particle size and PDI were recorded (Fig. 3B). Little changes were found in its size and PDI, indicating the good stability and dispersion in PBS.

The ion release behavior of MnO_2_@GA-Fe@CAI in PBS (pH 5.0, 6.5, and 7.4) was investigated. In an acidic PBS solution (pH 5.0), the accumulative release rate of Mn^2+^ and Fe^2+^from MnO_2_@GA-Fe@CAI reached about 51.13% (Fig. 3C) and 67.33% (Fig. 3D), respectively. Due to the breakage of the covalent bond between GA and Fe^3+^ under acidic conditions, the metal polyphenol network structure was disintegrated, releasing Mn^2+^ and Fe^2+^. Therefore, MnO_2_@GA-Fe@CAI nanoparticles had obvious acid-responsive release properties. As water-soluble metal ions, Mn^2+^ and Fe^2+^ could be excreted through the kidneys, so MnO_2_@GA-Fe@CAI was safe in the body.

### Cytotoxicity

The biocompatibility of nanoparticles was further examined through co-cultures with L929 cells and the relative cell viability was measured using a CCK-8 assay kit. As shown in Additional file 1: Fig. S4, most L929 cells survived under normal oxygen conditions, which was not significantly different from the control group.

**Fig. 4:**
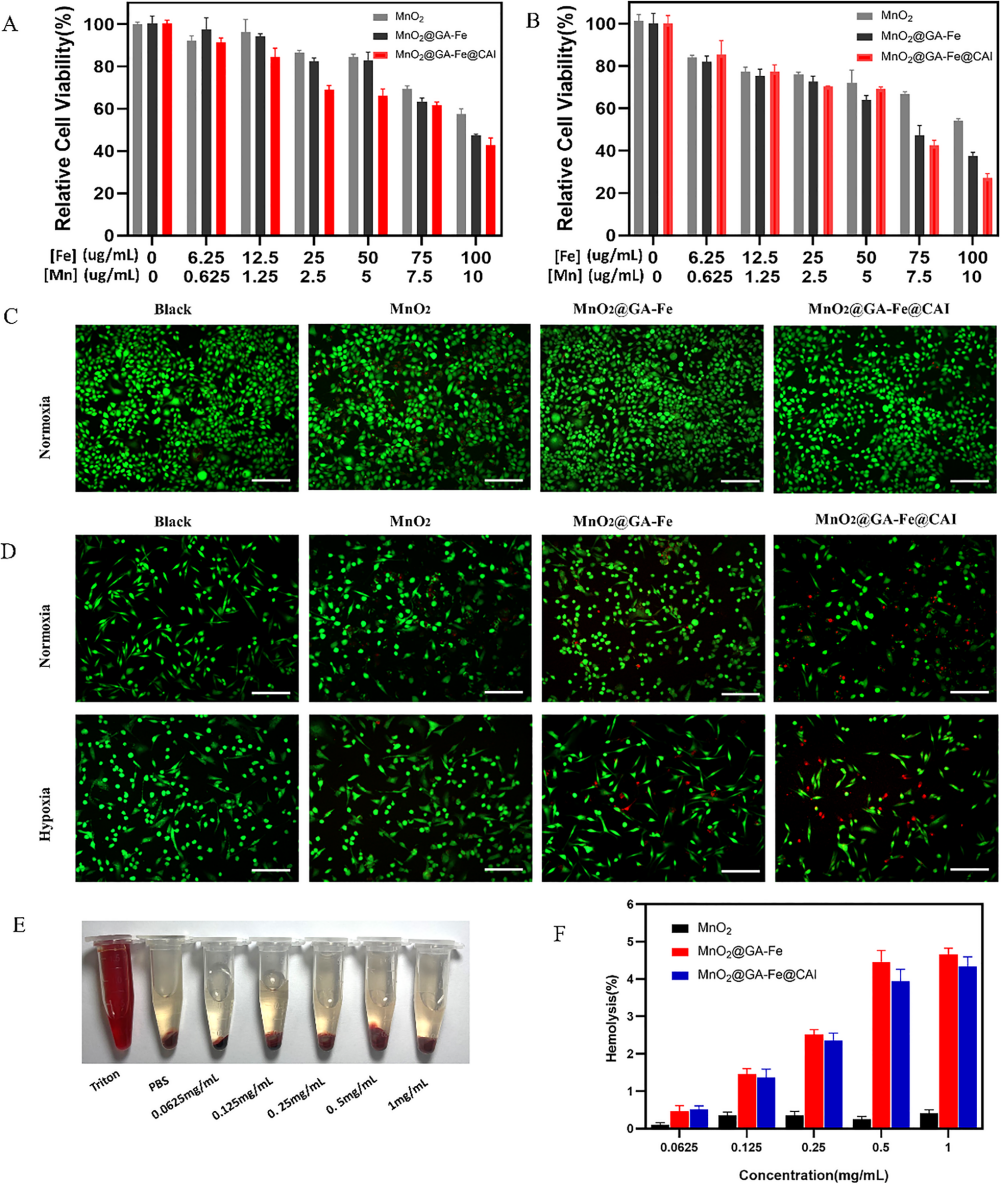
Relative cell viability after incubation with different concentrations of nanoparticles for 24 h. **A** MDA-MB-231 under normoxia, (**B**) MDA-MB-231 under hypoxia. Live/dead cell staining of (**C**) L929 and (**D**) MDA-MB-231 (scale: 100 μm). **E** Visual diagram of MnO_2_@GA-Fe@CAI hemolysis. **F** Hemolysis rate of different nanoparticles

The anti-tumor properties of MnO_2_@GA-Fe@CAI against MDA-MB-231 cells were also tested. CA IX on MDA-MB-231 cell membranes is highly expressed in a hypoxic environment [38]. Therefore, cells were cultured under normoxia and hypoxia to study the cytotoxicity of nanoparticles and calculate the IC50 value (Additional file 1: Table S1). The relative cell survival of MDA-MB-231 under normoxia decreased with increasing nanoparticle concentrations, showing a significant concentration-dependent manner (Fig. 4A). The cell survival rate under hypoxia was considerably lower than that under normoxia (Fig. 4B). For example, when Fe concentration reached 100 ug/mL, the relative cell survival rates under normoxia and hypoxia were 47.81 ± 2.23%, 27.12 ± 2.08%, respectively. And a similar trend was found in live/dead cell staining (Fig. 4C, D). The blood compatibility results showed that there was almost no red blood cell rupture, even when the highest concentration nanoparticles (1 mg/mL) were incubated with blood (Fig. 4E). The hemolysis rate was below the threshold of 5%, required for clinical application (Fig. 4F). At the same time, a strong absorption peak was observed in the cell supernatant of the positive control group (Triton X-100) (Additional file 1: Fig. S5), which indicated the hemolysis occurred. However, there was no significant difference among other experimental groups at the wavelength range of 480 ~ 680 nm, further supporting the biosafety of MnO_2_@GA-Fe@CAI.

### Intracellular ROS and GSH detection

**Fig. 5:**
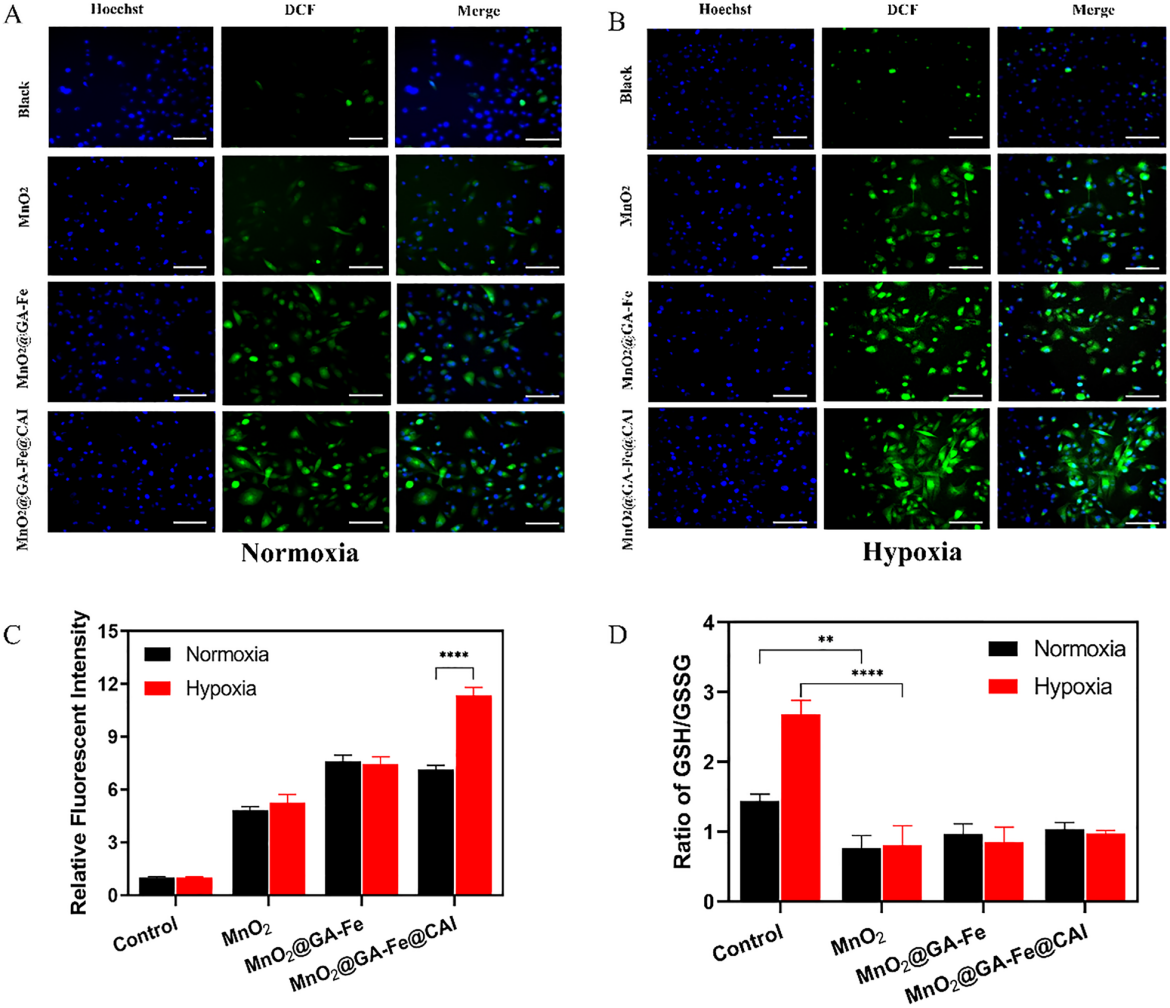
Detection of intracellular ROS in MDA-MB-231 cells after 8 h incubation with nanoparticles in (**A**) normoxia and (**B**) hypoxia (blue: nuclear, green: ROS, scale: 100 μm) (**C**) Relative fluorescence intensity of ROS (Mean ± SD, n = 3, **** p < 0.0001) (**D**) Ratio of GSH/GSSG (Mean ± SD, n = 3, **p < 0.01, **** p < 0.0001)

The ability of MnO_2_@GA-Fe@CAI to produce ROS was further investigated by the DCFH-DA probe. The DCFH-DA probe could freely cross the cell membrane and was hydrolyzed and oxidized by ROS to produce strong green fluorescence DCF [39]. As shown in Fig. 5A, B, a higher green fluorescence was observed in the MnO_2_@GA-Fe@CAI group than in the control group. In addition, compared with normoxia, there was strong ROS green fluorescence for MnO_2_@GA-Fe@CAI under hypoxia, indicating that it might further regulate the TME and increase the production of intracellular ROS. By semi-quantitative analysis of the relative fluorescence intensity, the fluorescence intensity of MDA-MB-231 cells treated with MnO_2_@GA-Fe@CAI under hypoxia was significantly higher than that of normoxia and control group (Fig. 5C). The above results indicated that the combined intervention by free Mn^2+^ and Fe^2+^ induced stronger intracellular ROS levels than single Mn^2+^. In addition, the Fenton reaction was further enhanced and more ROS was produced in tumor hypoxia TME.

As an antioxidant in cells, GSH could reduce ROS levels and chemical dynamics efficiency [40]. Therefore, the effects of nanoparticles on GSH levels in cells were evaluated by GSH and GSSG detection kits. After co-incubating nanoparticles with cells for 8 h, MnO_2_@GA-Fe@CAI decreased the ratio of GSH/GSSG by 28.6% and 63.73% under normoxia and hypoxia, respectively (Fig. 5D). MnO_2_@GA-Fe@CAI could convert GSH to GSSG in MDA-MB-231 cells, thus reducing GSH level in the cells. The decrease in GSH content could help to reduce the cell clearance of exogenous ROS, promote the occurrence of the Fenton reaction, and enhance the level of oxidative stress in tumor cells. Thus, bimetallic composite nanoparticles MnO_2_@GA-Fe@CAI could remodel the tumor microenvironment to lower endogenous GSH and improve the efficiency of Fenton.

### Mitochondrial membrane potential and apoptosis detection

**Fig. 6:**
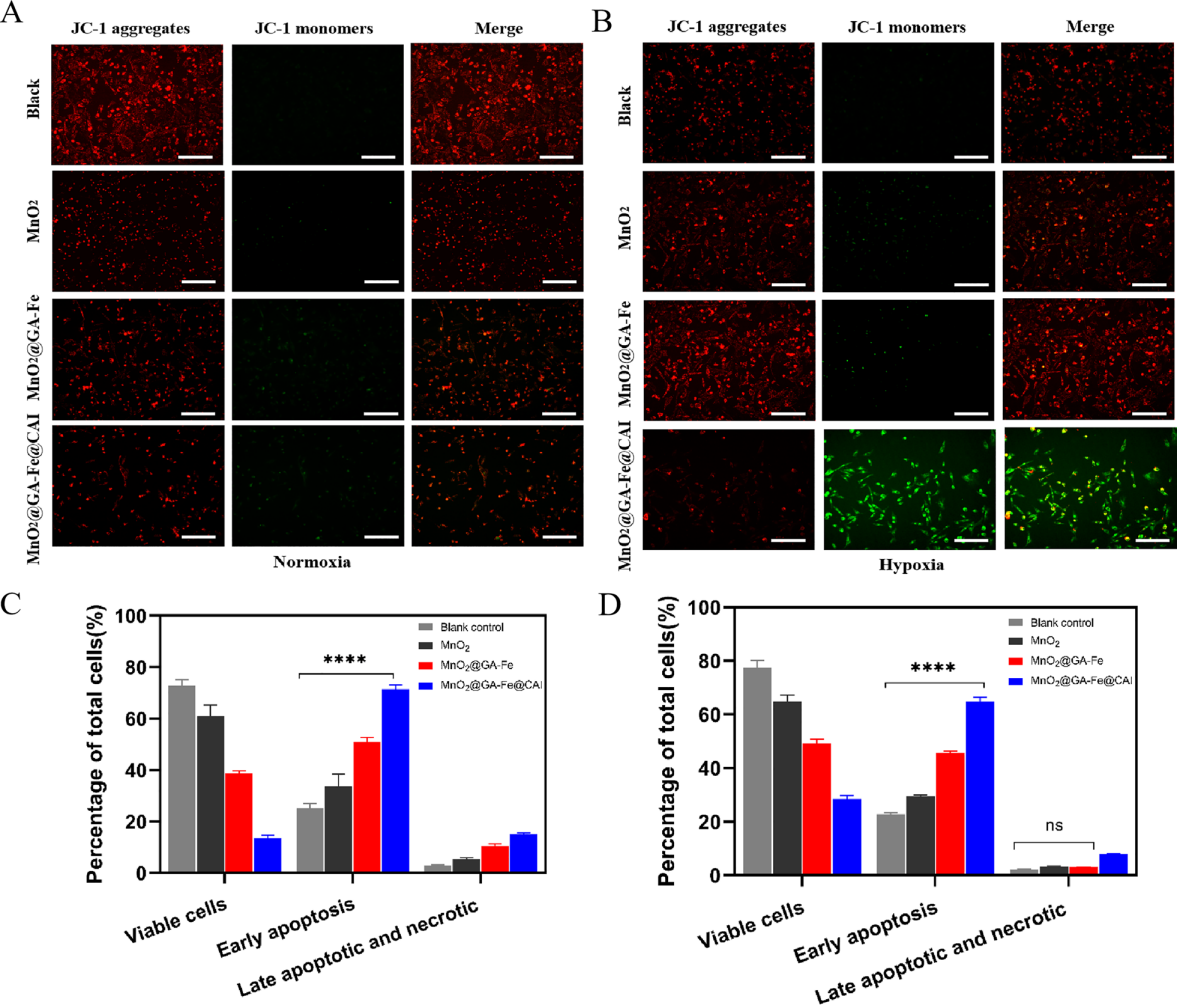
Mitochondrial membrane potential of MDA-MB-231 incubated with nanoparticles in (**A**) normoxia and (**B**) hypoxia (Red: JC-1 aggregates, Green: JC-1 monomers; scale: 100 μm). Early apoptosis ratio of MDA-MB-231 cells in (**C**) normoxia (**D**) hypoxia (n = 3, Mean ± SD, ****p < 0.0001)

The JC-1 probe was a mitochondrial potential probe to detect mitochondrial damage. The probe showed red fluorescence in standard mitochondrial membrane potential but was present as a green fluorescent monomer in damaged or depolarised mitochondrial membranes [41]. The effects of MnO_2_@GA-Fe@CAI on the mitochondrial membrane potential of MDA-MB-231 under normoxia (Fig. 6A) and hypoxia (Fig. 6B) were detected. Interestingly, MnO_2_@GA-Fe@CAI was able to significantly down-regulate FL2/FL1 under hypoxia, probably due to the sustained production of ROS by the Fenton reaction occurring in nanoparticles under hypoxia, which resulted in damage to the mitochondrial membrane and exacerbated the decrease in mitochondrial membrane potential (Additional file 1: Fig. S6).

Apoptosis levels of nanoparticles under normoxia (Additional file 1: Fig. S7 A) and hypoxia (Additional file 1: Fig. S7 B) were detected using flow cytometry. The different cell states were counted under normoxia (Fig. 6C), and MnO_2_@GA-Fe@CAI induced early apoptosis rate was 64.84 ± 1.66%. While under hypoxia, the particle induced a high rate of early apoptosis of 71.45 ± 1.70% (Fig. 6D). The results showed that MnO_2_@GA-Fe@CAI was able to increase the level of ROS in tumor cells and cause apoptosis under both normoxia and hypoxia and especial a strong ability under hypoxia.

### Research on cell acidization and cell migration

**Fig. 7:**
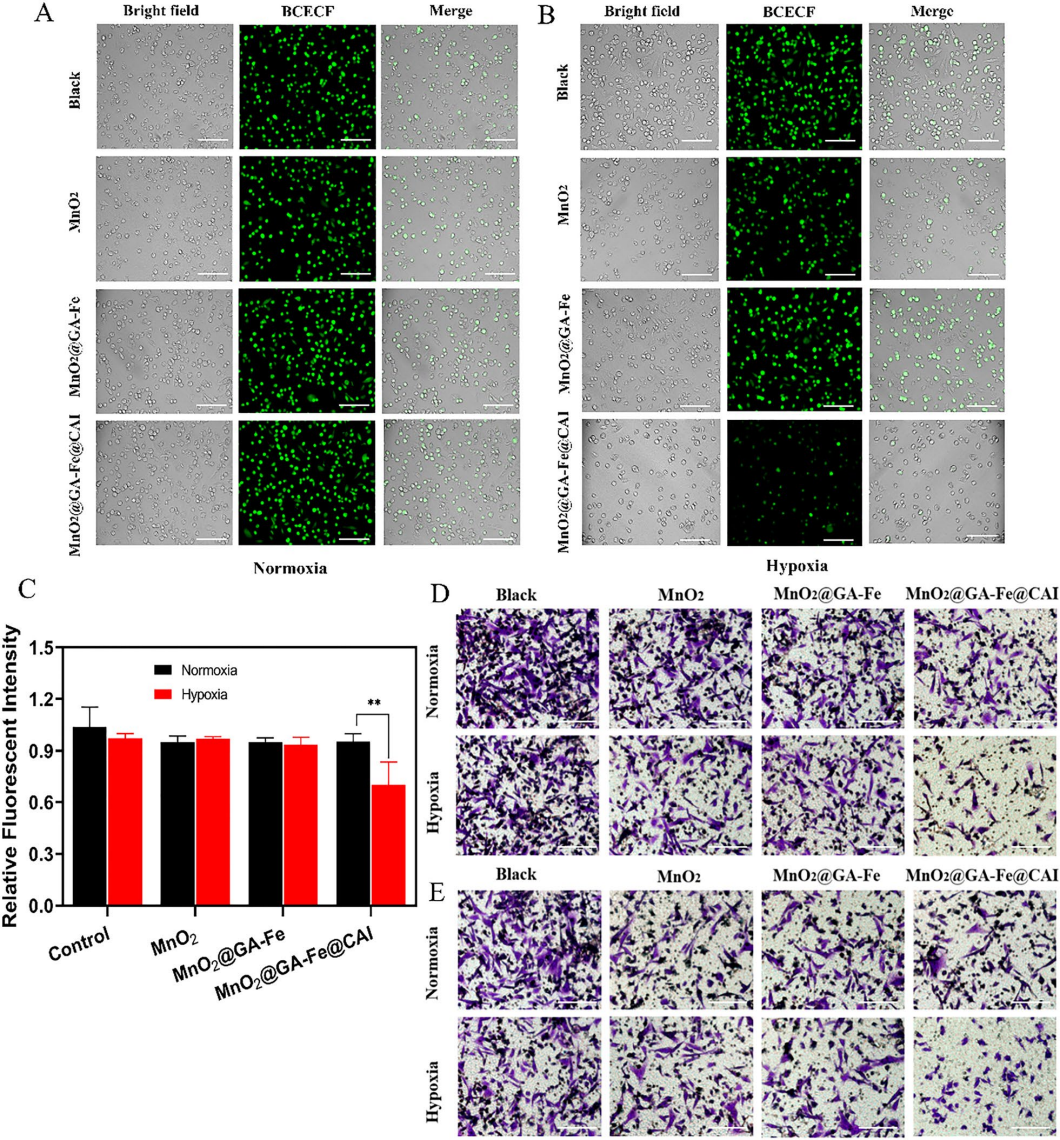
Results of intracellular acidification of MDA-MB-231 treated with nanoparticles under normoxia (**A**) and hypoxia (**B**) scale: 100 μm. **C** relative fluorescence intensity (Mean ± SD, n = 3, **p < 0.01). Migration (**D**) and invasion (**E**) of MDA-MB-231 after nanoparticles treated (scale: 100 μm)

The pH value in TME is about 6.5 ~ 7.0, while the optimal pH value for the Fenton reaction is 2 ~ 4 [42]. Therefore, lowering the pH value of TME is also an effective way to improve the efficiency of the Fenton reaction. BCECF can be excited to form green fluorescence at an appropriate pH value, and the higher the pH value, the higher the fluorescence intensity. We used the BCECF-AM fluorescent probe to explore the changes in endocellular pH value. Under normoxia, the fluorescence intensity of MDA-MB-231 cells slightly decreased after incubation with nanoparticles (Fig. 7A). However, under hypoxia, the fluorescence intensity of the MnO_2_@GA-Fe@CAI group was significantly lower than that of the other groups (Fig. 7B), which might be because CA IX was lowly expressed on the cell membrane under normoxia. By semi-quantitative analysis of fluorescence, the fluorescence intensity after MnO_2_@GA-Fe@CAI treatment was 0.92 and 0.72 times higher than that of the control under normoxia and hypoxia, respectively (Fig. 7C). It indicated that over-expression of CA IX in hypoxia could lead to a more potent inhibitory effect of MnO_2_@GA-Fe@CAI on CA IX, ultimately leading to intracellular acidification.

To further investigate the effect of MnO_2_@GA-Fe@CAI on the inhibition of TNBC metastasis, the behavior of tumor cells was studied by transwell cell migration and invasion assays. MnO_2_@GA-Fe@CAI could effectively suppress cell migration (Fig. 7D) and invasion (Fig. 7E) under normoxia and hypoxia. The inhibitory effect was more evident under hypoxia than under normoxia, which might be attributed to the excessive expression of CA IX under hypoxia. Thus, CAI may reduce the formation of extracellular HCO_3_^−^/H^+^ by inhibiting CA IX and causing acidic ions to accumulate in the cell. MnO_2_@GA-Fe@CAI ultimately inhibits the acidification of the cytoplasmic matrix environment, reducing the extent of tumor cell migration and invasion. Tumor pH-regulating proton pump inhibitors and protein inhibitors are capable of modulating proton pump activity to suppress lactate proton efflux and induce intracellular acidification. Proton pump inhibitors have shown promise in reconstructing the TME to facilitate the Fenton reaction. These findings highlight the therapeutic potential of such inhibitors for tumor treatment.

### In vivo treatment

**Fig. 8:**
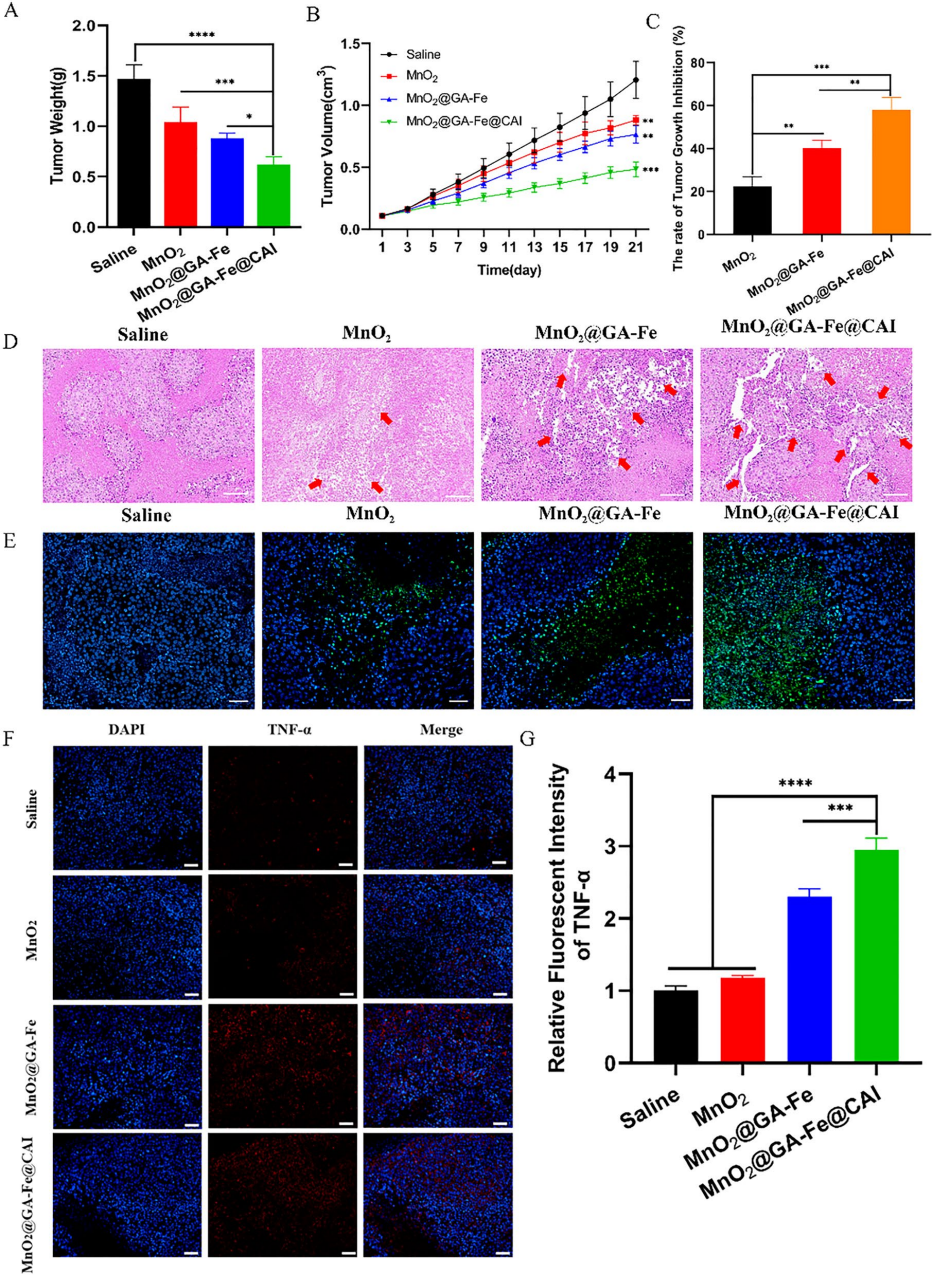
Tumor-bearing mice treated with nanoparticles for 21 days. **A** Tumor weight (n = 5, *p < 0.05, ***p < 0.001, ****p < 0.0001) (**B**) Tumor volume (n = 5, **p < 0.01, ***p < 0.001: significantly different from the Saline group.) (**C**) The rate of tumor growth inhibition (n = 5, **p < 0.01, ***p < 0.001) (**D**) H&E staining of tumor tissues (Red arrow: nuclear looseness, scale: 100 μm) (**E**) TUNEL apoptosis staining (Green fluorescence: apoptosis cells, scale: 100 μm) (**F**) TNF-α staining of tumor tissues in each group (scale: 100 μm) (**G**) Relative fluorescence intensity of TNF-α (***p < 0.001, ****p < 0.0001)

The anti-tumor performance of nanoparticles was further investigated on xenografted MDA-MB-B231 tumor-bearing mice. After 21 days of treatment, the results showed that all mice gained weight stably without adverse effects (Additional file 1: Fig. S8A). By analyzing the changes in tumor weight (Fig. 8A) and volume (Fig. 8B) during the treatment period, MnO_2_@GA-Fe@CAI treatment showed a clear trend of inhibition with lower tumor weight and smaller volume than that of control, as well as the higher tumor inhibition rates 58.09 ± 5.77% (Fig. 8C). Meanwhile, the images of extracted tumors after 21 days treated became small (Additional file 1: Fig. S8B), indicating the potential of MnO_2_@GA-Fe@CAI in inhibiting tumor growth. The distribution of the nanoparticles in mice after 0, 3, 6, 12, and 24 h of in situ administration was observed by the IVIS (Additional file 1: Fig. S8C). The results showed that DiD-MnO_2_@GA-Fe@CAI nanoparticles mainly aggregated at the tumor site. After 24 h, the tumor site still showed strong fluorescence, and some of the nanoparticles had already entered the liver, while other major organs did not show fluorescence (Additional file 1: Fig. S8D).

The H&E results showed that the number of tumor cells was reduced after MnO_2_@GA-Fe@CAI treatment, with red arrows indicating conditions such as loose arrangement and disappearance of nuclei, which exhibited the most severe degree of necrosis (Fig. 8D). In addition, TUNEL fluorescence staining was performed on each group of tumor tissues to detect the ability of nanoparticles to induce apoptosis, where green fluorescence in TUNEL indicates apoptosis. As shown in Fig. 8E, almost no green fluorescence was observed in the saline group; a small and faint fluorescence could be seen in the MnO_2_ groups. Among them, the MnO_2_@GA-Fe@CAI treated group showed bright green fluorescence, indicating many cells underwent apoptosis. To further evaluate the biosafety of the nanoparticles, H&E sections of major organs were used for further pathological evaluation. The results showed (Additional file 1: Fig. S9) that no significant damage and histological abnormalities or inflammatory lesions were observed in the heart, liver, spleen, lung and kidney in all groups. The biotoxicity of each nanoparticle-treated group was not significantly different from that of the saline group, indicating the nanoparticles had good biological safety in vivo.

TNF-α, as an important cytokine in cells, could induce apoptosis signal transduction through the death domain and kill tumor cells [43]. Thus, TNF-α fluorescence staining was performed on tumor tissues. The results showed that the tumor cells in the saline group hardly produced TNF-α red fluorescence. In contrast, the tumor tissues in MnO_2_@GA-Fe@CAI group had the strongest ability to induce TNF-α production among other groups (Fig. 8F). Relative fluorescence intensity of TNF-α results indicated that MnO_2_@GA-Fe@CAI could induce tumor cells apoptosis and enhancing the inhibitory effect on tumors (Fig. 8G). Combined with the previous results at the cellular level, MnO_2_@GA-Fe@CAI nanoparticles were possible ways to achieve efficient tumor inhibition by depleting GSH in tumors, acidifying and remodeling the TME of tumors, achieving ROS amplification, and prompting apoptosis of tumor cells.

## Conclusions

In conclusion, the multifunctional bimetallic composite nanoparticles MnO_2_@GA-Fe@CAI were designed with ideal biocompatibility. The nanoparticles lowered intracellular pH and endogenous GSH by remodeling the tumor microenvironment, increasing Fenton activity and achieving an efficient CDT effect catalyzed by bimetals. The MnO_2_@GA-Fe@CAI nanoparticles could reach the tumor site. CAI inhibited the activity and expression of CA IX enzymes, decreased the pH value in the tumor microenvironment, and provided favorable conditions for the Fenton reaction. Then, the nanoparticles penetrated the tumor, and the MPN structure was gradually depolymerized due to the weak pH acidity in TME. MnO_2_ could convert GSH into GSSG and reduce the content of antioxidant substances in tumor cells, while Mn^4+^ was reduced to Mn^2+^ and GA ameliorated Fe^3+^ to Fe^2+^. Ultimately, Mn^2+^ and Fe^2+^ could act as catalysts in the Fenton reaction to produce OH from H_2_O_2_ and achieve efficient synergistic treatment of TNBC. So MnO_2_@GA-Fe@CAI could regulate the tumor microenvironment in multiple ways, enhance the efficiency of intracellular CDT, effectively inhibit the invasion and metastasis of triple-negative cancer cells, and achieve further treatment of TNBC.

### Supplementary Information

**Additional file 1: Table S1.** IC50 values of MDA-MB-231and L929 cells after incubation for 24 h with MnO_2_,MnO_2_@GA-Fe and MnO_2_@GA-Fe@CAI （n=3, x±SD）. **Fig S1.** (A) EDS element mapping of MnO_2_@GA-Fe@CAI nanoparticles: Fe (blue), Mn (red), S (yellow) and O (green). (B) EDS of MnO_2_@GA-Fe@CAI nanoparticles. **Fig S2.** The FT-IR spectra of nanoparticles. **Fig S3.** UV-vis absorption spectra of (A)PAH and KMnO_4_ solution and MnO_2_ NPs (B) MnO_2_@GA-Fe and MnO_2_@GA-Fe@CAI. **Fig S4.** Relative cell viability of L929 cells incubated with different concentrations of nanoparticles for 24 h. **Fig S5.** UV-vis absorption spectra of MDА-MB-231 supernatant treated with nanoparticles (positive control: Triton X-100, negative control: PBS). **Fig S6.** The semi-quantitative calculation results of JC-1 after MDА-MB-231 co-cultured with nanoparticles for 8 h. (FL2: JC-1 aggregates, FL1: JC-1 monomers, Mean ± SD, n=3, ***p < 0.001). **Fig S7.** Nanoparticles induced apoptosis of MDА-MB-231 after 12 h incubation under (A) normoxia and (B) hypoxia. **Fig S8.** (A) Body weight of tumоr-bearing mice (B) A photo of tumor at 21 days. (C) The time-dependent biodistribution of DiD-MnO_2_@GA-Fe@CAI after in intratumoral injection. (D) Biodistribution of DiD-MnO_2_@GA-Fe@CAI in the main organs and tumor after 24 h post-intratumoral injection. **Fig S9.** H&E stаining оf vital оrgаns оf tumоr-beаring mice 21 dаys after treatment (scale: 100 μm).

## Data Availability

No data was used for the research described in the article.
