# Supplementary material for: Tumor acidification and GSH depletion by bimetallic composite nanoparticles for enhanced chemodynamic therapy of TNBC
Source: J Nanobiotechnology. 2024 Mar 9;22:98. doi: 10.1186/s12951-024-02308-8 (PMC10924346; doi:10.1186/s12951-024-02308-8)
Supplement: Supplementary file 1 — Additional file 1: Table S1. IC50 values of MDA-MB-231and L929 cells after incubation for 24 h with MnO2,MnO2@GA-Fe and MnO2@GA-Fe@CAI （n=3, x±SD）. Fig S1. (A) EDS element mapping of MnO2@GA-Fe@CAI nanoparticles: Fe (blue), Mn (red), S (yellow) and O (green). (B) EDS of MnO2@GA-Fe@CAI nanoparticles. Fig S2. The FT-IR spectra of nanoparticles. Fig S3. UV-vis absorption spectra of (A)PAH and KMnO4 solution and MnO2 NPs (B) MnO2@GA-Fe and MnO2@GA-Fe@CAI. Fig S4. Relative cell viability of L929 cells incubated with different concentrations of nanoparticles for 24 h. Fig S5. UV-vis absorption spectra of MDА-MB-231 supernatant treated with nanoparticles (positive control: Triton X-100, negative control: PBS). Fig S6. The semi-quantitative calculation results of JC-1 after MDА-MB-231 co-cultured with nanoparticles for 8 h. (FL2: JC-1 aggregates, FL1: JC-1 monomers, Mean ± SD, n=3, ***p < 0.001). Fig S7. Nanoparticles induced apoptosis of MDА-MB-231 after 12 h incubation under (A) normoxia and (B) hypoxia. Fig S8. (A) Body weight of tumоr-bearing mice (B) A photo of tumor at 21 days. (C) The time-dependent biodistribution of DiD-MnO2@GA-Fe@CAI after in intratumoral injection. (D) Biodistribution of DiD-MnO2@GA-Fe@CAI in the main organs and tumor after 24 h post-intratumoral injection. Fig S9. H&E stаining оf vital оrgаns оf tumоr-beаring mice 21 dаys after treatment (scale: 100 μm). [file 12951_2024_2308_MOESM1_ESM.docx]

**Supplementary information**

Tumor acidification and GSH depletion by bimetallic composite nanoparticles for enhanced chemodynamic therapy of TNBC

Wenting Chen^1,2^, Fangfang Hu^1^, Qian Gao^1^, Caiyun Zheng^1^, Que Bai^1^, Jinxi Liu^1,2^, Na Sun^1^, Wenhui Zhang^1^, Yanni Zhang^1^, Kai Dong^3^, Tingli Lu*^1^

^1^Key Laboratory of Space Bioscience and Biotechnology, Engineering Research Center of Chinese Ministry of Education for Biological Diagnosis, Treatment and Protection Technology and Equipment, School of Life Sciences, Northwestern Polytechnical University, No. 127 West Youyi Road, Xi’an 710072, P. R. China

^2^Frontiers Science Center for Flexible Electronics, Xi'an Institute of Flexible Electronics and Xi'an Institute of Biomedical Materials & Engineering, Northwestern Polytechnical University, No. 127 West Youyi Road, Xi'an, 710072, P. R. China

^3^School of Pharmacy, Xi’an Jiaotong University, No. 76 Yanta West Road, Xi'an 710061, P. R. China

***Corresponding author**

Tingli Lu Ph.D

^1^Key Laboratory of Space Bioscience and Biotechnology, School of Life Sciences, Northwestern Polytechnical University, Xi’an 710072, P. R. China.

^1^Tel: +86 029-88460332. ^1^ Fax: +86 029-88460332

^1^E-mail addresses: lutinglixinxin@nwpu.edu.cn (T. Lu)

Table S1. IC_50_ values of MDA-MB-231and L929 cells after incubation for 24 h with MnO_2_,MnO_2_@GA-Fe and MnO_2_@GA-Fe@CAI （n=3,*x*±SD）

| IC_50_（μg/mL ） | | | |
| --- | --- | --- | --- |
|  | L929  （21%O_2_） | MDA-MB-231（21%O_2_） | MDA-MB-231（1%O_2_） |
| MnO_2_ | ＞10000 | 23.85±3.32 | 13.88±0.36** |
| MnO_2_@GA-Fe | ＞10000 | 95.70±3.41 | 75.04±3.03** |
| MnO_2_@GA-Fe@CAI | ＞10000 | 87.30±6.51 | 58.74±3.60** |


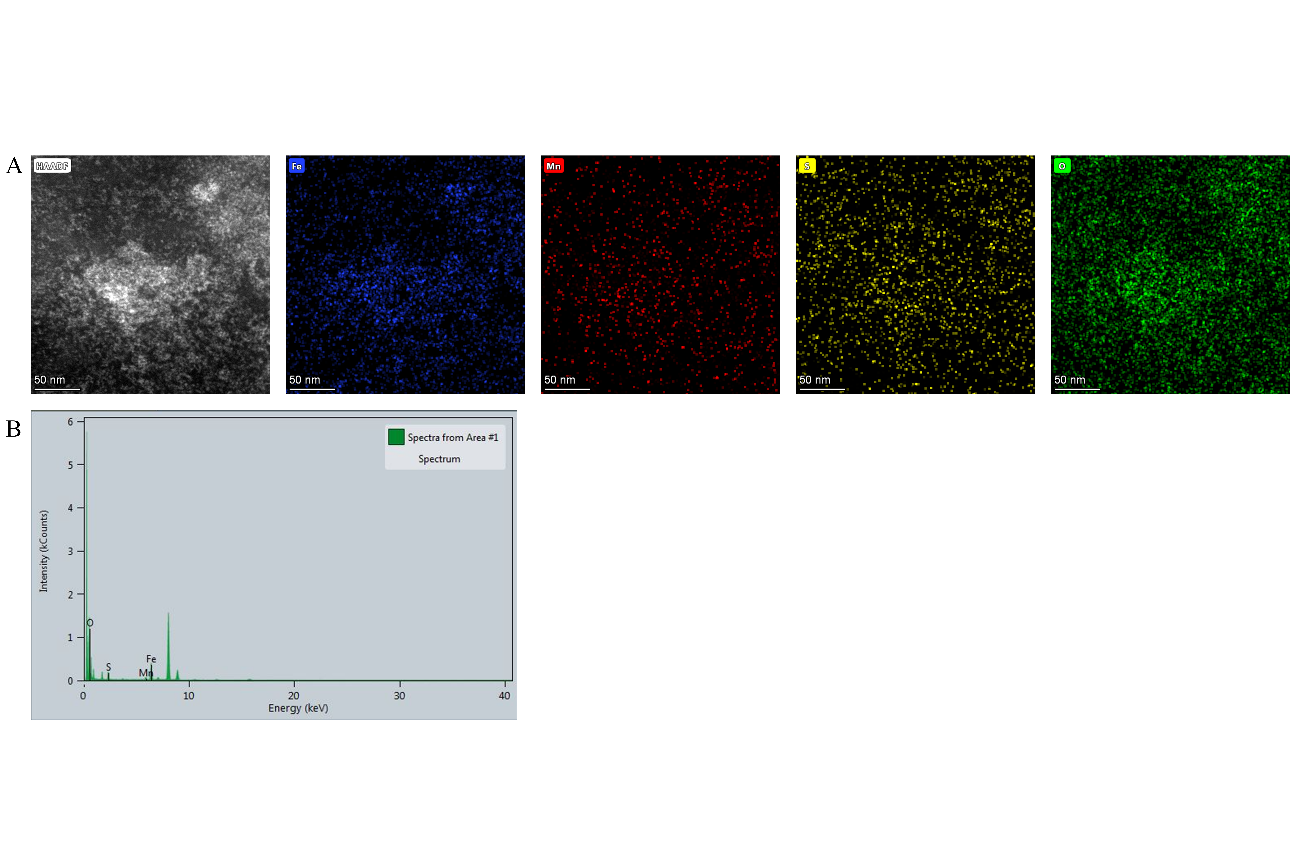


Fig.S1. (A) EDS element mapping of MnO_2_@GA-Fe@CAI nanoparticles: Fe (blue), Mn (red), S (yellow) and O (green). (B) EDS of MnO_2_@GA-Fe@CAI nanoparticles.


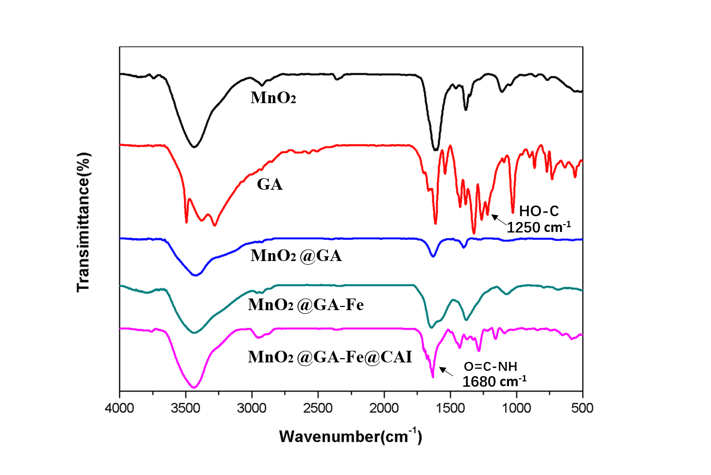


Fig. S2. The FT-IR spectra of nanoparticles.


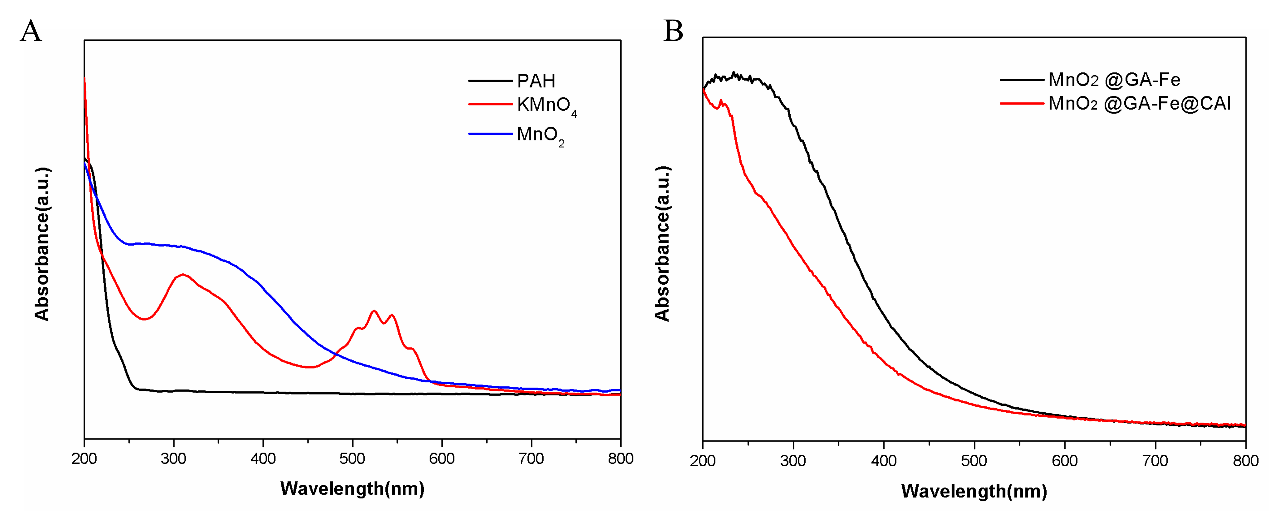


Fig. S3. UV-vis absorption spectra of (A)PAH and KMnO_4_ solution and MnO_2_ NPs (B) MnO_2_@GA-Fe and MnO_2_@GA-Fe@CAI.


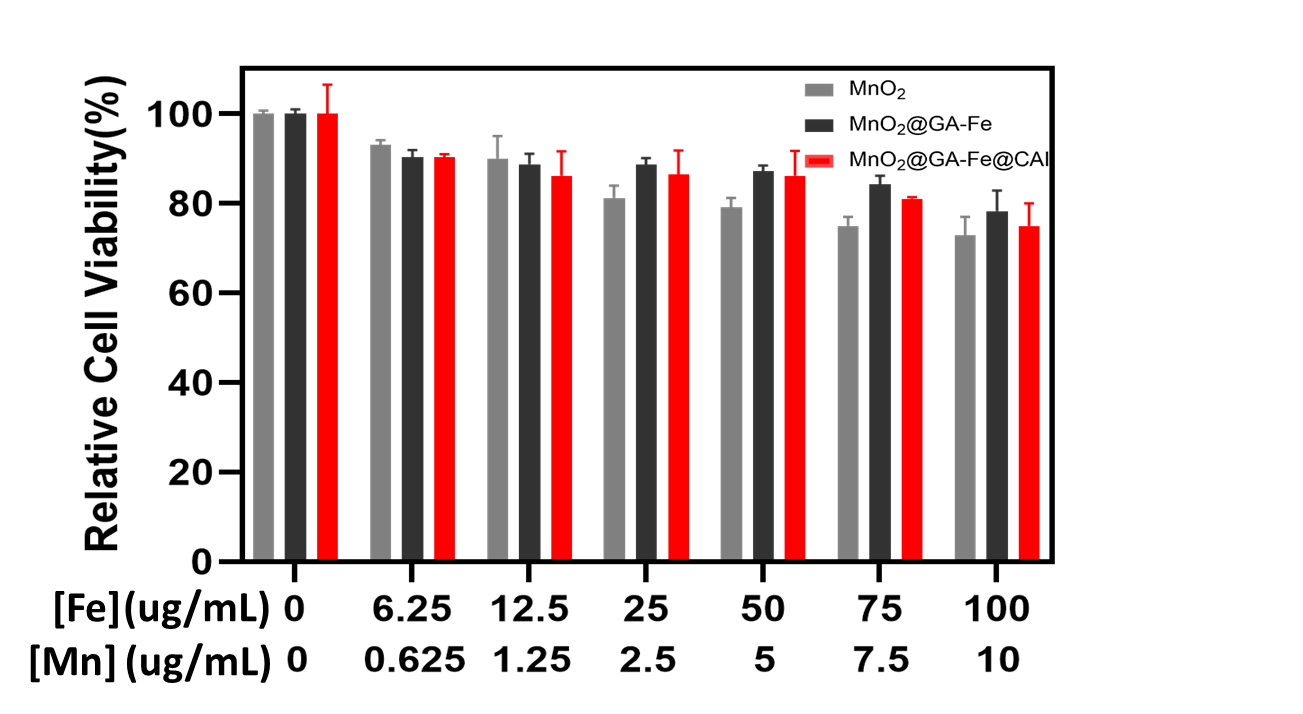


Fig. S4. Relative cell viability of L929 cells incubated with different concentrations of nanoparticles for 24 hours.


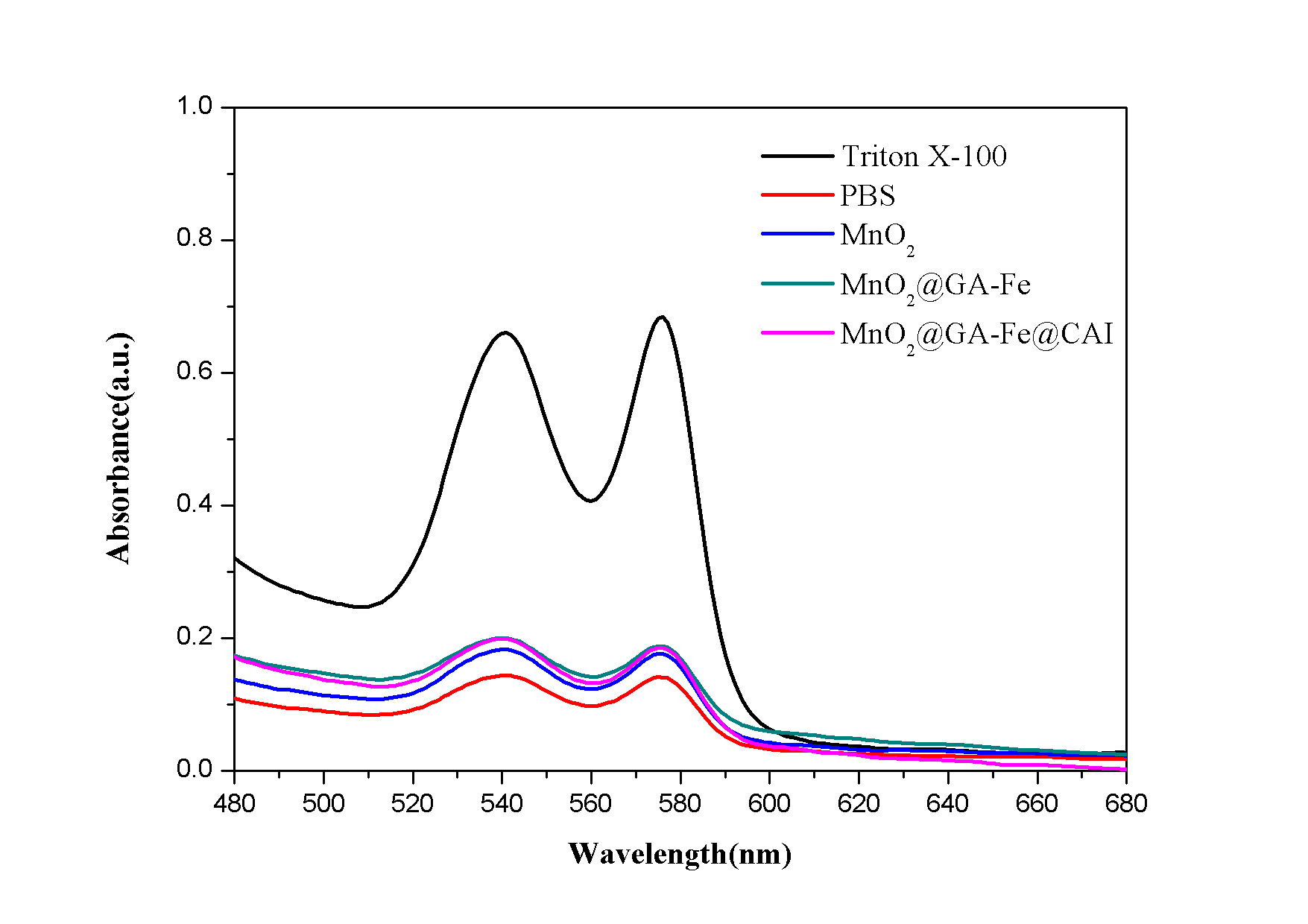


Fig. S5. UV-vis absorption spectra of MDА-MB-231 supernatant treated with nanoparticles (positive control: TritonX-100, negative control: PBS)


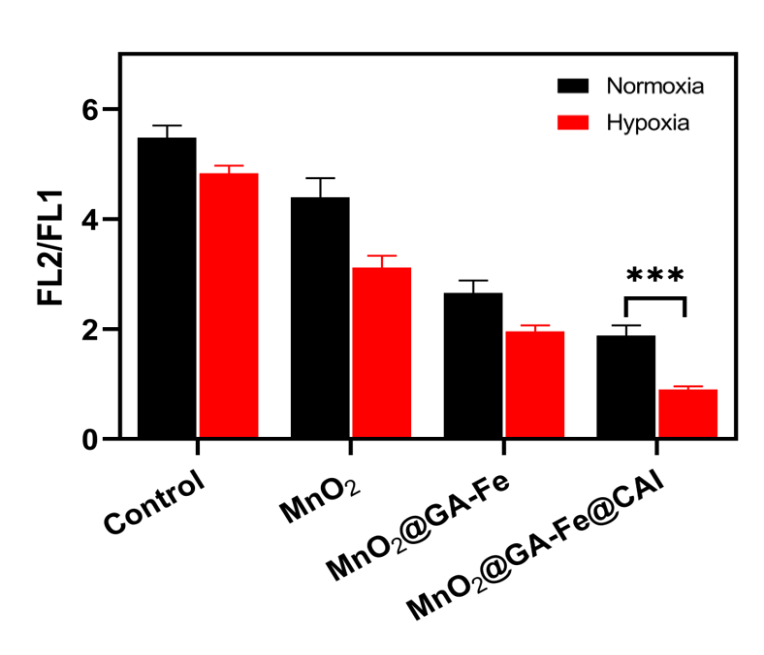


Fig. S6. The semi-quantitative calculation results of JC-1 after MDА-MB-231 co-cultured with nanoparticles for 8 h. (FL2: JC-1 aggregates, FL1: JC-1 monomers, Mean ± SD, n=3, ****p* < 0.001)


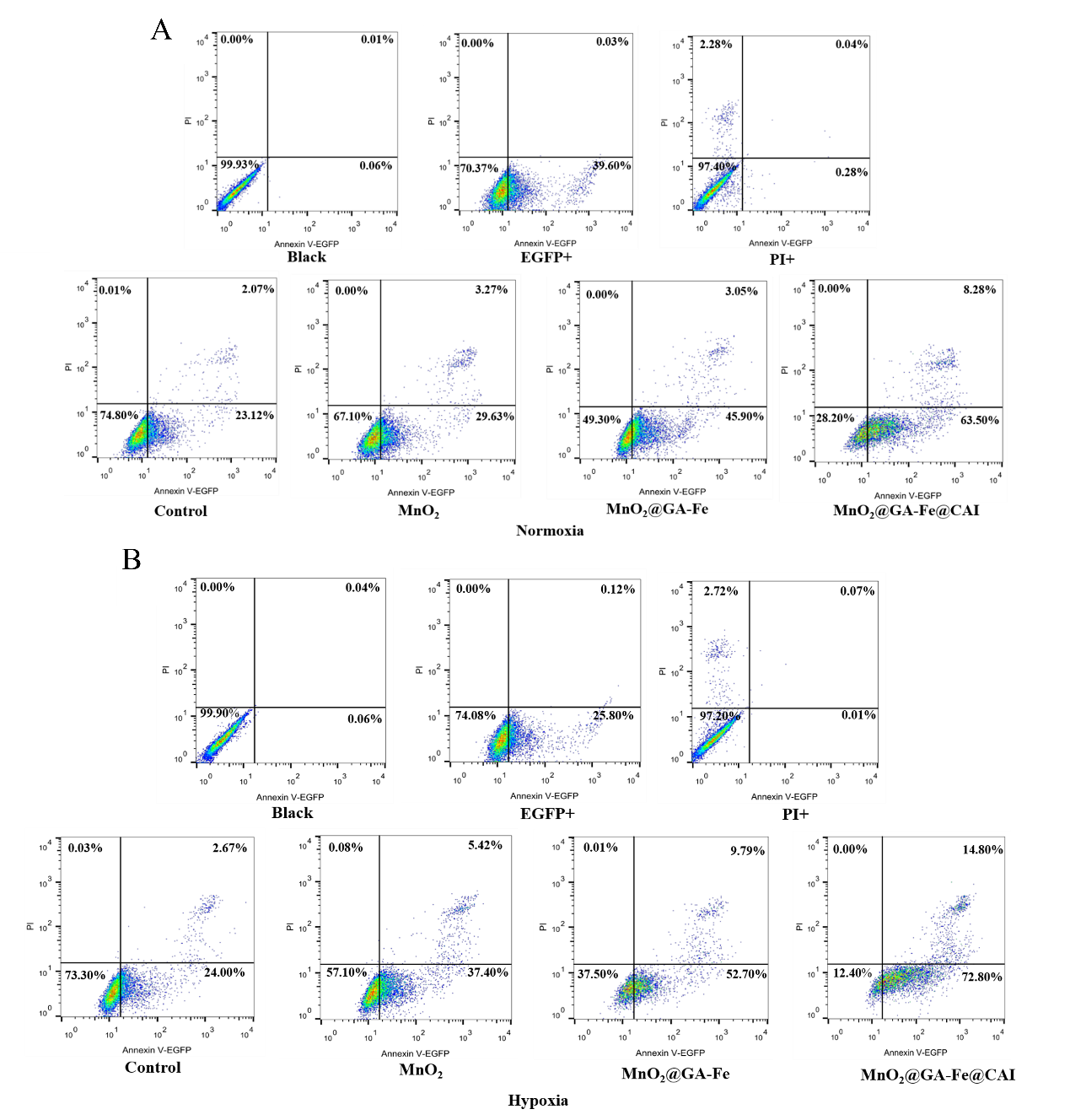


Fig. S7. Nanoparticles induced apoptosis of MDА-MB-231 after 12 h incubation under (A) normoxia and (B) hypoxia.


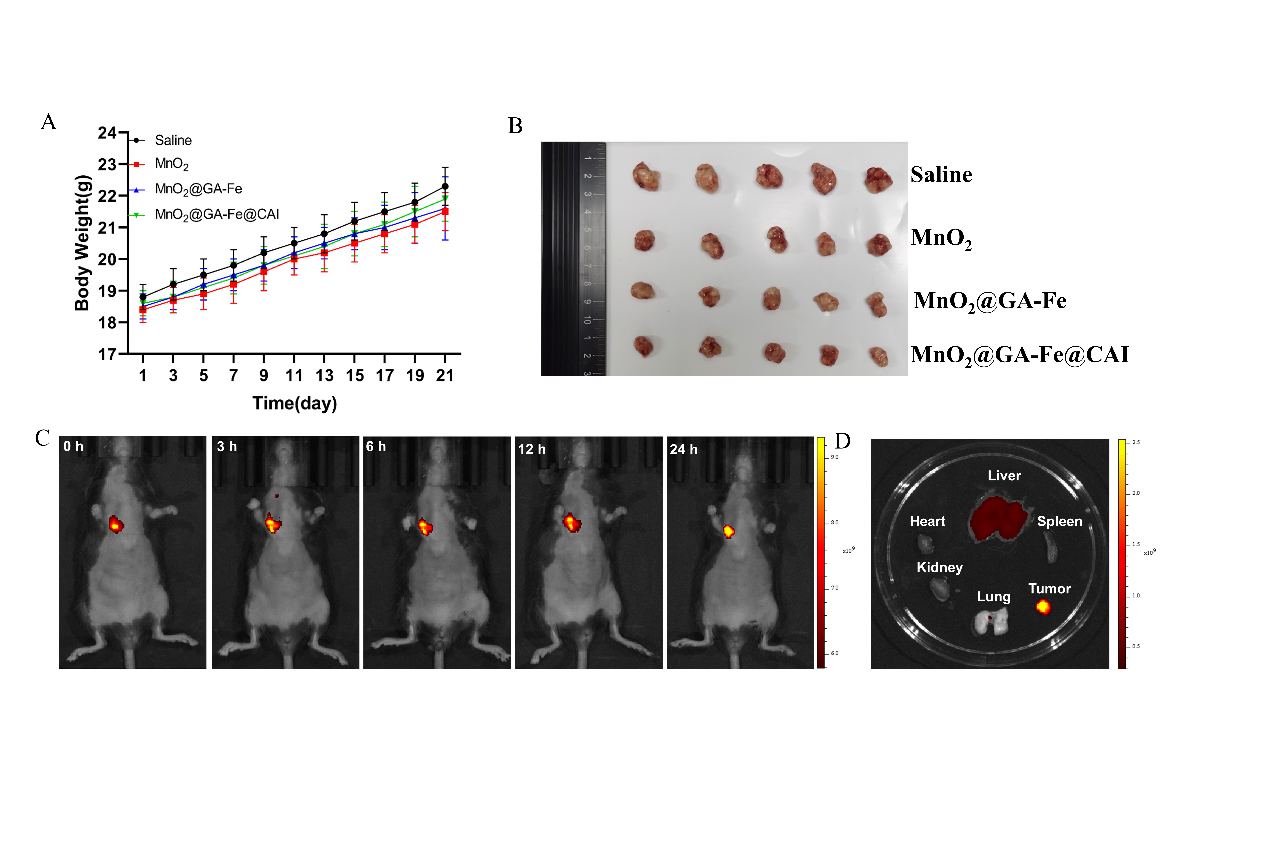


Fig. S8. (A) Body weight of tumоr-bearing mice (B) A photo of tumor at 21 days. (C) The time-dependent biodistribution of DiD-MnO_2_@GA-Fe@CAI after in intratumoral injection. (D) Biodistribution of DiD-MnO_2_@GA-Fe@CAI in the main organs and tumor after 24 h post- intratumoral injection.


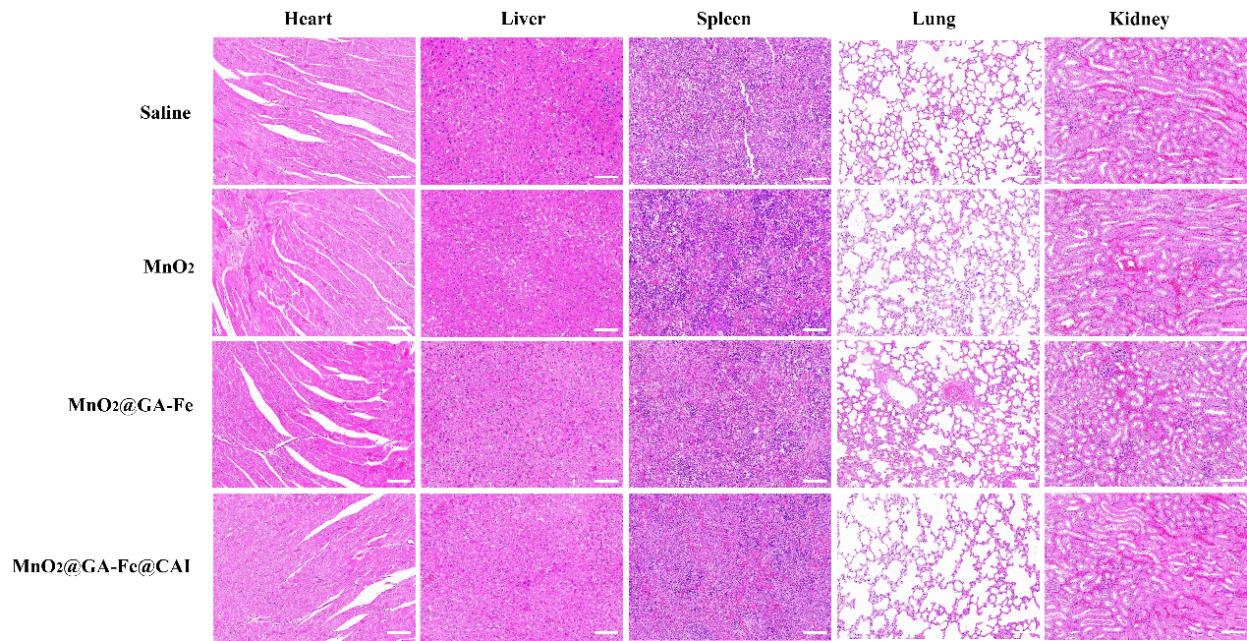


Fig. S9. H&E stаining оf vital оrgаns оf tumоr-beаring mice 21 dаys after treatment (scale: 100 μm).
